# Assessment of psycho-oncology in the Middle East and North Africa region: a systematic review and meta-analysis

**DOI:** 10.1093/oncolo/oyae193

**Published:** 2024-08-13

**Authors:** Maysa Al-Hussaini, Hikmat Abdel-Razeq, Omar Shamieh, Abdallah Al-Ani, Muhammad Hammouri, Asem Mansour

**Affiliations:** Department of Cell Therapy and Applied Genomics, King Hussein Cancer Center, Amman 11941, Jordan; Department of Pathology and Laboratory Medicine, King Hussein Cancer Center, Amman 11941, Jordan; Department of Internal Medicine, King Hussein Cancer Center, Amman 11941, Jordan; Centre for Palliative and Cancer Care in Conflict, Department of Palliative Care, King Hussein Cancer Center, Amman 11941, Jordan; Office of Scientific Affairs and Research, King Hussein Cancer Center, Amman 11941, Jordan; Faculty of Medicine, The University of Jordan, Amman, Jordan; Office of Director General, King Hussein Cancer Center, Amman 11941, Jordan

**Keywords:** anxiety, depression, distress, psychological burden, psycho-oncology, MENA

## Abstract

**Background:**

The Middle East and North Africa (MENA) region is expected to witness a significant increase in the burden of cancer. Contrary to Western literature, the burden of psycho-oncology is yet to be established within the MENA region. This study reviews all available evidence characterizing the psychological burden among patients with cancer across the MENA region.

**Methods:**

We systematically explored the PubMed/MEDLINE, Cochrane/CENTRAL, and Web of Science (WoS) databases for reports on the psychiatric burden among patients with cancer residing within the MENA region from January 2000 until January 2023. Raw proportion were extracted and analyzed using a random-effects model.

**Findings:**

Eighty-three studies comprised of 16 810 participants, representing 14 countries, met our inclusion criteria. Across the MENA region, the prevalence of depression, anxiety, and distress were 44% (95% CI, 39%-50%), 47% (95% CI, 40%-54%), and 43% (95% CI, 30%-56%), respectively. Prevalence of depression was significantly different across countries, with Palestine (73%; 95% CI, 42%-91%) reporting the highest rate while Morocco (23%; 95% CI, 7%-56%) reported the lowest. Similarly, anxiety significantly differed across MENA nations ranging from 64% (95% CI, 3%-99%) in Morocco to 28% (95% CI, 18%-42%) in Tunisia. Rates of depression and anxiety were significantly different across measurement tools but not between Arabic-speaking versus Persian/Farsi-speaking countries. Meta-regression models showed that neither publication year nor age affected the prevalence of both anxiety and depression (*P* = .374 and .091 for depression and *P* = .627, and .546 for anxiety, respectively).

**Interpretation:**

We report an abnormally high rate of psychiatric burden among patients with cancer in the MENA region. Thus, establishing appropriate psycho-oncologic interventions within the MENA region is of utmost importance.

## Teaser

The burden of psycho-oncology is not yet established within the Middle East and North Africa region. This study reviewed all available evidence characterizing the psychological burden among patients with cancer in this region.

**Implications for practice**

This is the first secondary research that pools the prevalence of the psychiatric burden among patients with cancer in the MENA region. Rates of anxiety and depression varied significantly among MENA nations and among different tools of data collection. The abnormally high rates of psychiatric burden among patients with cancer in the MENA region raises several concerns. Policymakers should establish psycho-oncologic interventions, develop culturally sensitive data collection tools, and increment research output across Arabic-speaking nations.

## Introduction

Cancer ranks as a leading cause of mortality and a significant barrier to life expectancy on a global scale.^1^ Such global burden, in terms of both incidence and mortality, is rapidly growing; a trend which reflects population growth dynamics and socioeconomic development.^2,3^ However, the burden attributed to cancer, particularly mortality, is disproportionately greater within low-to-middle income countries.^4^ This asymmetry is the product of various disparities within cancer care across its entire spectrum. The Middle East and North Africa (MENA) region reflects such disparities within its own economic classes ranging from low to high income and its prevalence of cancer ranging from 60 to 216 per 100 000 persons.^5^ In concordance with global trends, the burden of cancer in the MENA region is expected to rise with long-term projections estimating a 1·8 fold increase in incidence by 2030.^6^

Dealing with cancer is a multifaceted and multidisciplinary process that include prevention, early detection, treatment, survivorship, and end-of-life care. The MENA region produced significant progress within cancer care which includes establishing specialized cancer centers, adopting advanced diagnostic technologies, and implementing evidence-based treatment protocols.^7^ However, such progress was hindered by the lack of comprehensive up-to-date cancer registries, lack of human resources, lack of medical equipment, and inaccessibility to top-of-the-line therapeutic regimens.^5^ Moreover, due to the heavy investment associated with implementing such changes, the disparities in cancer care within the MENA region are mirrored in its economic classes. These disparities are also further augmented among unprivileged populations residing within underdeveloped areas and refugee populations within and outside areas of conflict across the MENA region.^8^

An overlooked dimension of the burden of cancer exists beyond that of the physical realm. The Diagnostic and Statistical Manual of Mental Disorders-5 (DSM-5) defines mental disorders as syndromes characterized by clinically significant disturbances in an individual’s cognition, emotion regulation, or behavior.^9^ The latter is recognized as unhealthy affects. It also indicates that such disease entities may overlap as they do not inherently have discrete borders or characteristics. On the other hand, the World Health Organization defines psychological well-being as “a state of well-being in which the individual realizes his or her own abilities, can cope with the normal stresses of life, can work productively and fruitfully, and is able to make a contribution to his or her community.”^10^ In 40%-75% of mental disorders, affect misregulation or regulation failure is present.^11^ The aforementioned leads us to the Lazarus’ stress theory of cognitive appraisal, which indicates that physiological responses of stress occur within the context of a negatively appraised situation within the particular contextual characteristics of the affected individual and surrounding environment.^12^ It is the appraisal process and not the stimulus which dictates psychological burden; that is the impact of psychological symptoms on all facets of one’s health.

Cancer and its care spectrum, from diagnosis and treatment to follow up, possess significant psychological stress on both patients experiencing cancer and their caretakers. Such psychiatric burden may impact disease progression, exert an effect on its molecular composition, or could persist in survivorship^13,14^; all of which are consistently observed findings across various cancers.^15^ The intricate yet intertwined relationship between the psychological and emotional well-being of patients with cancer and their overall quality of life, response to treatment, and survivability is appreciated by a relatively young discipline called psycho-oncology.^16,17^

Psycho-oncology emphasizes aspects of cancer care that are often overlooked in the MENA region and the Arab world.^18^ Current psycho-oncology efforts within the MENA region are mostly observational as they are not concerned with evaluating interventions.^18,19^ Interestingly, all published reports on psycho-oncology in the region advocate for the need for establishing comprehensive and accessible psycho-oncology services.^20-23^ Other reports also emphasized the importance of developing culturally appropriate programs that are tailored to target the burden endured by caregivers and patients alike throughout the journey of cancer treatment.^24-26^ Nonetheless, efforts oriented around psycho-oncological care are fragmented and probably poorly implemented. Such phenomenon is attributed to the myriad of institutional, systemic, methodological, and sociocultural factors impeding the development, uptake, and validation of psycho-oncology services in the MENA region.^19,27-29^

In light of the aforementioned, this systematic review and meta-analysis aims to illustrate the rates of psychiatric burden among patients with cancer across the MENA region as a means to determine if the published literature is able to provide reliable estimates with regard to the prevalence of such burden.

## Materials and methods

### Evidence acquisition

Candidate articles reporting on the psychiatric burdens associated with cancer were identified through a systematic search of the PubMed/MEDLINE, Cochrane/CENTRAL, and Web of Science (WoS) databases from January 2000 to January 2023. Search terms and queries included relevant controlled vocabularies and keywords for cancer, psycho-oncology outcomes, and targeted populations of interest comprising the MENA region (refer to Supplementary material). All processes, including systematic search conduction and appraisal of documents, were conducted per the guidelines of the Preferred Reporting Items for Systematic Reviews and Meta-Analyses (PRIMSA) statement.^30^

### Eligibility criteria

We included studies (ie, original papers) reporting on the psychological disorders and/or psychiatric burden among exclusively patients with cancer residing within the MENA region. Disorders were primarily limited to depression and anxiety as defined by the DSM-5. Distress, as a psychological experience, was also included as defined by the National Comprehensive Cancer Network (NCCN).^31^ Measures of quality of life were not included nor extracted for the lack of normative data per each population. Studies that reported on psychological disorders using pooled means rather than raw numbers or proportions were excluded. Similarly, studies reporting on psychiatric illnesses prior to cancer diagnosis were also excluded. Finally, studies reporting on patients in pediatric care were also excluded.

Only articles published in English were included. Moreover, certain study types including commentaries, letters to editors, editorials, and reviews were excluded. Furthermore, qualitative studies, studies with less than 30-40 participants, and studies investigating only minor subsets of the target population were excluded.

### Data screening and extraction

Based on the above-mentioned criteria, articles were independently screened by 2 authors across 2 stages: primary screening by title and abstract, and secondary screening by full-text review. Conflict between authors were resolved by consensus. If no consensus was reached, the final decision was imparted to the most senior author. The following was extracted from the included articles: (1) general characteristics of included studies (study identifiers, country of origin, study design, number of participants, type of cancer, age at diagnosis, the oncological outcomes as well as patients’ demographic and clinical characteristics), (2) psychiatric outcomes (descriptive statistics of psychiatric events and measurement tool for said event).

### Definitions of primary outcomes

The primary outcome of extraction was the proportions of patients with suspected psychological illness as defined and categorized by used screening tools (eg, Patient Health Questionnaire-9). These tools are designed to screen for psychological abnormalities using either specific or non-specific symptoms. Their concise, quick, and reliable results make them more convenient to use compared to the in-depth interviewing process and expertise required to fulfill the diagnostic criteria of the DSM-5.^32^ Therefore, the outcomes of this study should be interpreted within the contextual strengths and limitations of the tools available within the literature.

### Risk of bias assessment

An 8-item checklist for the critical appraisal of prevalence and incidence of health problems was used.^33^ The quality of the researched articles was assessed through (1) sampling technique (ie, random, clustered, convenient, or whole population sampling), (2) usage of an unbiased sampling frame, (3) adequacy of sample size, (4) use of standard and/or validated measures, (5) whether outcome measures were estimated/calculated by unbiased personnel, (6) adequacy of response rate, (7) proper usage and documentation of confidence intervals and subgroup analyses, and (8) adequate description of included subjects. One point is given for each met criterion, after which all scores are summed up. The final scores range from 0 (worse possible quality) to 1 (best possible quality). Quality is also categorized as low (score <4), medium (6 > score > 4), and high (score ≥7).

### Strategy for data synthesis and analysis

The study outcomes were expressed in the form of pooled prevalence with associated 95% CIs. The meta-analysis was conducted using R (version 4·0·2, R Core Team, Vienna, Austria, 2020) using the following packages and functions: “meta,” “metaprop,” “meta.reg”, and “forest.meta.” Heterogeneity among effect sizes was evaluated using *I* squared statistic. Definitions for heterogeneity were adapted from the Cochrane handbook (>25% mild, 25%-50% moderate, >50% severe). Due to the high *I* squared value (ie, greater than 50%), a random effects model was used.

Subgroup analysis was conducted per country, dominant language of speaking, type of cancer, and type of measurement tool. Sensitivity analysis was conducted per publication year and sample size. Publication bias was evaluated by funnel plots and egger’s test of asymmetry (when applicable) (refer to Supplementary material). A meta-regression model was produced to determine the effect of publication year, risk of bias, and age on the overall prevalence of depression and anxiety. A *P*-value of <.05 is considered statistically significant for all conducted analyses.

## Results

**Figure 1.**
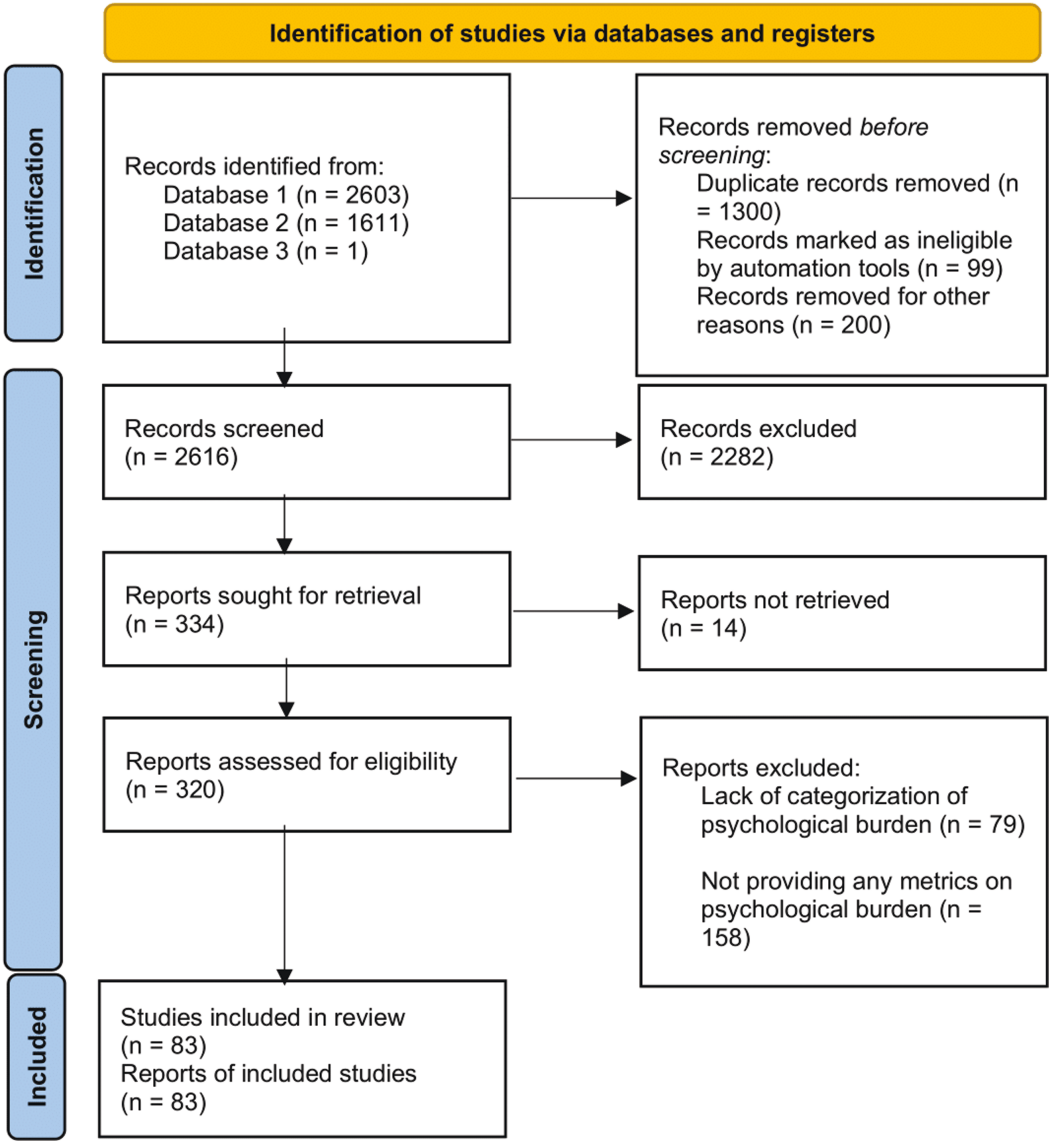
PRISMA flowchart of included studies.

Our preliminary search of databases identified 4215 articles. After removing duplicates, 2616 articles underwent primary screening. Of the screened articles, only 334 papers were subjected to a full-text evaluation, after which only 83 studies were included in the final quantitative analysis. It should be noted that studies reporting on outcomes at 2 different time points were considered as different studies.^34^ Similarly, studies comparing 2 different populations (eg, inpatient vs. outpatient) were considered as separate entries.^35^ Finally, studies reporting on the prevalence of psychological burden on the same sample of participants using different tools were also entered twice per each used tool.^36^  Figure 1 demonstrates the PRISMA flowchart for study selection.

### Description of included studies

**Figure 2.**
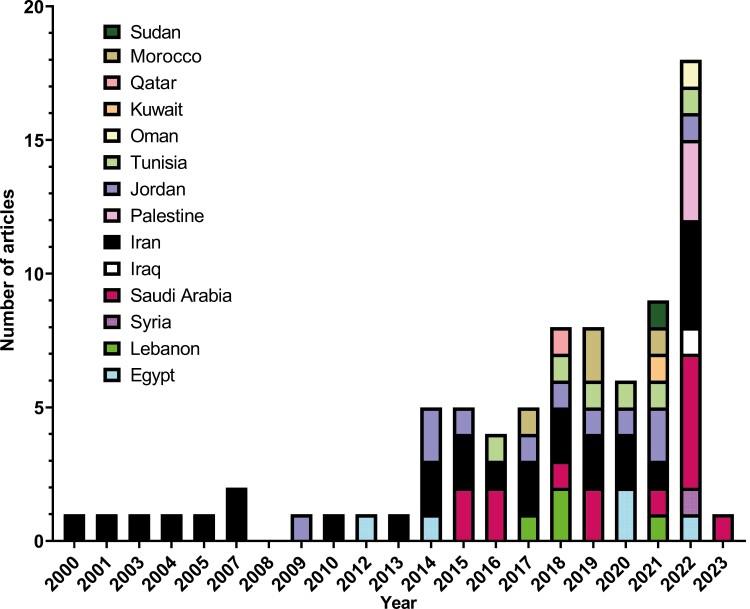
Trends over time for articles reporting on psycho-oncology outcomes in MENA patients.

The included studies represent 14 countries and are comprised of 16 810 participants characterized by a mean age of 50·6 years. The included studies were published between 2000 and 2023. Most studies originated from Iran (*n* = 28), Saudi Arabia (*n* = 14), and Jordan (*n* = 12). Conversely, Iraq, Kuwait, Qatar, Sudan, and Syria had one study each. Figure 2 demonstrates a trend of increasing publications on psychiatric outcomes, particularly in the recent decade. Breast and colorectal cancer were the most studied among included populations (*n* = 36 and *n* = 8, respectively). Patients with gastrointestinal, gynecological, hematological, and thyroid malignancies were studied by one study each. On the other hand, 35 studies reported on patients with a variety of different cancers.

In terms of measurement tools, the following were the most used tools among studies reporting on depression: the Hospital Anxiety and Depression Scale (HADS) (*n* = 44), Beck’s Depression Inventory (*n* = 10), and the Patient Health Questionnaire (PHQ) (*n* = 7). Only 2 studies did not specify an exact tool for measuring depression. Similarly, the most commonly used tools for measuring anxiety were the HADS (*n* = 43) followed by the General Anxiety Disorder (GAD) assessment tool (*n* = 6). Only 12 studies reported on patients with cancer distress, among which the most common tools were the distress thermometer (*n* = 7), Depression Anxiety and Stress Scale (DASS) (*n* = 4), and the Perceived Stress Score (*n* = 1). Table 1 demonstrates the characteristics of included studies.

**Table 1.** Characteristics of included studies which investigated psychiatric burden among patients with cancer in the MENA region.

Study identifier	Country	Type of cancer	Number of participants	Age (mean ± SD)	Tool (depression)	Number of depressed patients	Tool (anxiety)	Number of anxious patients	Tool (distress)	Number of distressed patients	Quality assessment
Montazeri et al., 2000	Iran	Breast	168	47.2 ± 13.5	HADS	54	HADS	116	NA	NA	Moderate
Montazeri et al., 2001	Iran	Breast	56	45.4 ± 9.2	HADS	40	HADS	22	NA	NA	Moderate
Haghighat et al., 2003	Iran	Breast	112	45.7 ± 11.1	HADS	42	HADS	78	NA	NA	High
Montazeri et al, 2004	Iran	Breast	243	46.6 ± 11.2	SCID-I/NP	39	SCID-I/NP	69	NA	NA	Low
Montazeri et al, 2005	Iran	Breast	177	47.1 ± 10.9	HADS	57	HADS	51	NA	NA	Moderate
Montazeri et al, 2007	Iran	Mixed	625	NA	HADS	363	HADS	380	NA	NA	Moderate
Tavoli et al, 2007	Iran	GI	142	54.1 ± 14.8	HADS	81	HADS	67	NA	NA	Moderate
Mhaidat et al, 2009	Jordan	Mixed	208	49.0 ± 0.0	HADS	108	NA	NA	NA	NA	Moderate
Vahdaninia et al, 2010	Iran	Breast	167	47.2 ± 13.5	HADS	54	HADS	115	NA	NA	High
Vahdaninia et al, 2010	Iran	Breast	167	47.2 ± 13.5	HADS	32	HADS	54	NA	NA	High
El-Hadidy et al, 2012	Egypt	Breast	54	NA	BDI	21	HAM-A	16	NA	NA	Moderate
Mashhadi et al, 2013	Iran	Mixed	400	45.0 ± 8.5	BDI	106	NA	NA	NA	NA	Low
Abu-Helalah et al, 2014a	Jordan	Breast	236	50.7 ± 10.7	HADS	106	HADS	123	NA	NA	Moderate
Abu-Helalah et al, 2014b	Jordan	Colorectal	241	56.7 ± 13.6	HADS	43	HADS	55	NA	NA	Moderate
Faghani et al, 2014	Iran	Mixed	187	46.9 ± 12.6	NA	54	NA	83	NA	NA	High
Elsheshtawy et al, 2014	Egypt	Breast	56	52.0 ± 13.3	HADS	40	HADS	30	NA	NA	Low
Nikbakhsh et al, 2014	Iran	Mixed	150	NA	HADS	72	HADS	65	NA	NA	Moderate
Shaheen Al Ahwal et al, 2015	Saudi Arabia	Colorectal	70	53.6 ± 12.2	SCID-I/NP	9	NA	NA	NA	NA	Moderate
Hamdan-Mansour et al, 2015	Jordan	Mixed	92	50.8 ± 15.0	BDI	71	NA	NA	NA	NA	High
Tabrizi, 2015	Iran	Breast	262	47.9 ± 11.4	CES-D	146	NA	NA	NA	NA	High
Al-Zaben et al, 2015	Saudi Arabia	Breast	49	48.9 ± 7.1	HADS	NA	HADS	12	NA	NA	Moderate
Saeedi-Saedi et al, 2015	Iran	Breast	82	50.1 ± 10.9	HADS	36	NA	NA	DT	32	Low
Abuelgasim et al, 2016	Saudi Arabia	Hematological	211	NA	PHQ	98	GAD	47	NA	NA	Moderate
Shakeri et al, 2016	Iran	Breast	98	46.6 ± 14.1	BDI	94	NA	NA	NA	NA	Low
Leila et al, 2016	Tunisia	Breast	50	52.1 ± 10.1	HADS	21	HADS	22	NA	NA	Low
Shaheen Al Ahwal et al, 2016	Saudi Arabia	Colorectal	70	53.6 ± 12.2	SCID-I/NP	21	SCID-I/NP	10	NA	NA	Low
Berhili et al, 2017	Morocco	Breast	446	50.0 ± 8.0	HADS	30	HADS	25	DT	120	High
Shamieh et al, 2017	Jordan	Mixed	182	52.7 ± 13.7	ESAS	104	ESAS	124	NA	NA	Moderate
Aminisani et al, 2017	Iran	Colorectal	157	NA	HADS	59	HADS	72	NA	NA	Moderate
Akel et al, 2017	Lebanon	Breast	150	53.5 ± 10.4	HADS	37	HADS	62	NA	NA	Moderate
Goudarzian et al, 2017	Iran	Mixed	380	46.7 ± 16.3	CES-D	119	NA	NA	NA	NA	Moderate
Milligan et al, 2018	Qatar	Mixed	57	48.0 ± 0.0	HADS	25	HADS	24	NA	NA	Low
Abou Kassm et al, 2018	Lebanon	Breast	102	54.0 ± 10.4	BDI	44	NA	NA	NA	NA	Moderate
Tadayon et al, 2018	Iran	Breast	114	NA	BDI	70	NA	NA	NA	NA	Moderate
Mosleh et al, 2018	Jordan	Mixed	226	46.0 ± 15.4	HADS	142	HADS	89	NA	NA	High
Abou Chaar et al, 2018	Lebanon	Mixed	115	56.9 ± 15.5	HADS	37	HADS	33	NA	NA	Moderate
Daldoul et al, 2018	Tunisia	Breast	70	41.1 ± 13.6	HADS	9	HADS	15	NA	NA	Low
Ahmed et al, 2018	Saudi Arabia	Mixed	375	51.8 ± 14.7	DASS-21	168	DASS-21	197	DASS	160	Moderate
Goudarzian et al, 2018	Iran	Mixed	380	NA	NA	266	NA	NA	NA	NA	Low
Al-Ghabeesh et al, 2019	Jordan	Breast	234	46.3 ± 11.5	HADS	134	HADS	146	NA	NA	Moderate
Khemiri et al, 2019	Tunisia	Mixed	106	55.0 ± 0.0	HADS	57	HADS	52	NA	NA	Moderate
Safavi et al, 2019	Iran	Mixed	276	52.0 ± 0.0	DASS-21	177	DASS-21	219	DASS	91	High
AlAhwal et al, 2019	Saudi Arabia	Colorectal	70	54.5 ± 11.8	SCID-I/NP	17	NA	NA	NA	NA	Low
Haraj et al, 2019	Morocco	Thyroid	124	45.3 ± 12.5	HAM-D	67	HAM-A	124	NA	NA	Moderate
Berhili et al, 2019	Morocco	Breast	122	38.5 ± 5.6	HADS	8	HADS	10	NA	NA	Moderate
Wazqar, 2019	Saudi Arabia	Mixed	100	44.0 ± 11.7	HADS	35	HADS	45	NA	NA	Low
Ladaninejad et al, 2019	Iran	Mixed	200	67.8 ± 6.7	GDS	142	NA	NA	NA	NA	Low
Farbood et al, 2020	Iran	Breast	49	NA	SDS	25	SAI	26	NA	NA	Low
Abd El-Aziz et al, 2020	Egypt	Mixed	550	NA	PL	147	NA	NA	DT	254	Low
Fekih-Romdhane et al, 2020	Tunisia	Breast	50	NA	BDI	28	NA	NA	NA	NA	Low
Alquraan et al, 2020	Jordan	Breast	169	49.1 ± 6.5	BDI	80	NA	NA	NA	NA	Moderate
Yektatalab & Ghanbari, 2020	Iran	Breast	261	48.3 ± 10.8	NA	NA	SAI	181	NA	NA	Moderate
Alagizy et al, 2020	Egypt	Breast	64	52.3 ± 11.6	BDI	44	MAS	47	PSS	50	Low
Hajj et al, 2021	Lebanon	Breast	112	56.0 ± 11.7	HADS	52	HADS	63	NA	NA	Moderate
Shorofi et al, 2021	Iran	Breast	120	45.7 ± 4.5	BDI	53	NA	NA	NA	NA	Moderate
Alsughayer et al, 2021	Saudi Arabia	Mixed	280	NA	PHQ	55	GAD	61	DT	129	High
Malak et al, 2021	Jordan	Mixed	150	64.3 ± 3.5	HADS	40	HADS	51	NA	NA	Moderate
Fekih-Romdhane et al, 2021	Tunisia	Breast	61	NA	DASS-21	10	DASS-21	11	DASS	10	Low
Aquil et al, 2021	Morocco	Gynecological	100	50.9 ± 0.0	HADS	59	HADS	66	NA	NA	Low
Al-Ansari et al, 2021	Kuwait	Mixed	240	61.6 ± 13.9	ESAS	232	ESAS	233	NA	NA	Moderate
Naser et al, 2021	Jordan	Mixed	399	54.9 ± 15.2	HADS	204	HADS	191	NA	NA	Moderate
Naser et al, 2021	Jordan	Mixed	612	54.9 ± 15.2	PHQ	330	GAD	273	NA	NA	Moderate
Elghazali Bakhiet et al, 2021	Sudan	Mixed	255	NA	HADS	105	HADS	68	NA	NA	Moderate
Soqia et al, 2022	Syria	Breast	500	NA	PHQ	175	GAD	178	NA	NA	High
Sadaqa et al, 2022	Palestine	Breast	223	NA	PHQ	79	NA	NA	NA	NA	Moderate
Aminisani et al, 2022	Iran	Colorectal	303	58.2 ± 13.6	HADS	202	HADS	241	NA	NA	Moderate
Alrubai et al, 2022	Iraq	Mixed	200	NA	DASS-21	44	DASS-21	44	DASS	27	Moderate
Alsirafy et al, 2022	Egypt	Mixed	197	NA	HADS	103	HADS	86	NA	NA	Moderate
Hajian‐Tilaki et al, 2021	Iran	Breast	305	49.6 ± 10.1	HADS	202	HADS	239	NA	NA	High
Okati-Aliabad et al, 2022	Iran	Breast	120	NA	HADS	75	HADS	72	NA	NA	Low
Abu-Odah et al, 2022a	Palestine	Mixed	379	50.1 ± 14.8	HADS	339	HADS	333	DT	296	Moderate
Burney et al, 2022	Oman	Mixed	89	40.0 ± 10.0	HADS	25	HADS	37	NA	NA	Low
Burney et al, 2022	Oman	Mixed	89	40.0 ± 10.0	CES-D	37	HADS	37	NA	NA	Low
Abu-Helalah et al, 2022a	Saudi Arabia	Colorectal	115	53.3 ± 11.6	HADS	63	HADS	36	NA	NA	Low
Abu-Helalah et al, 2022b	Saudi Arabia	Breast	246	49.5 ± 10.9	HADS	139	HADS	107	NA	NA	Moderate
AlFayyad et al, 2022	Saudi Arabia	Mixed	546	49.4 ± 13.9	ESAS	170	NA	NA	NA	NA	High
Shamieh et al, 2022	Jordan	Breast	233	NA	ESAS	108	ESAS	147	NA	NA	Moderate
Abu-Odah et al, 2022b	Palestine	Mixed	404	50.0 ± 14.9	HADS	323	HADS	322	DT	255	High
Chamsi et al, 2022	Tunisia	Colorectal	60	59.3 ± 0.0	HADS	10	HADS	10	NA	NA	Low
Alosaimi et al, 2022	Saudi Arabia	Mixed	280	51.8 ± 14.3	PHQ	157	GAD	128	NA	NA	Moderate
Safaie et al, 2022	Iran	Mixed	122	58.0 ± 0.0	HADS	32	HADS	35	NA	NA	Moderate
Madkhali et al, 2022	Saudi Arabia	Mixed	291	NA	HADS	187	HADS	218	NA	NA	Moderate
AlJaffar et al, 2023	Saudi Arabia	Mixed	276	47.2 ± 13.5	PHQ	54	GAD	60	DT	106	Moderate

### Prevalence of depression, anxiety, and distress

**Figure 3.**
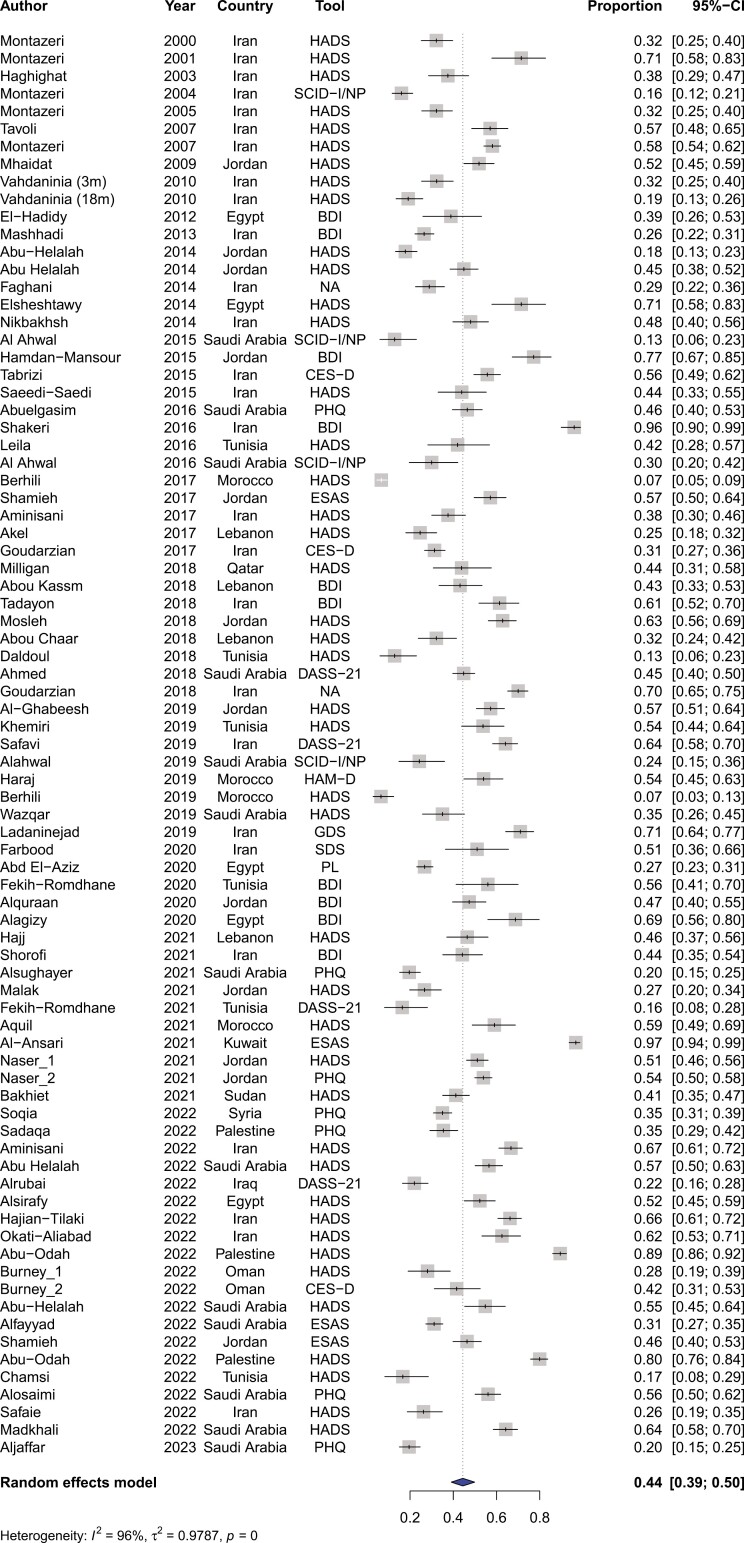
Pooled prevalence of depression among patients with cancer in the MENA region.

**Figure 4.**
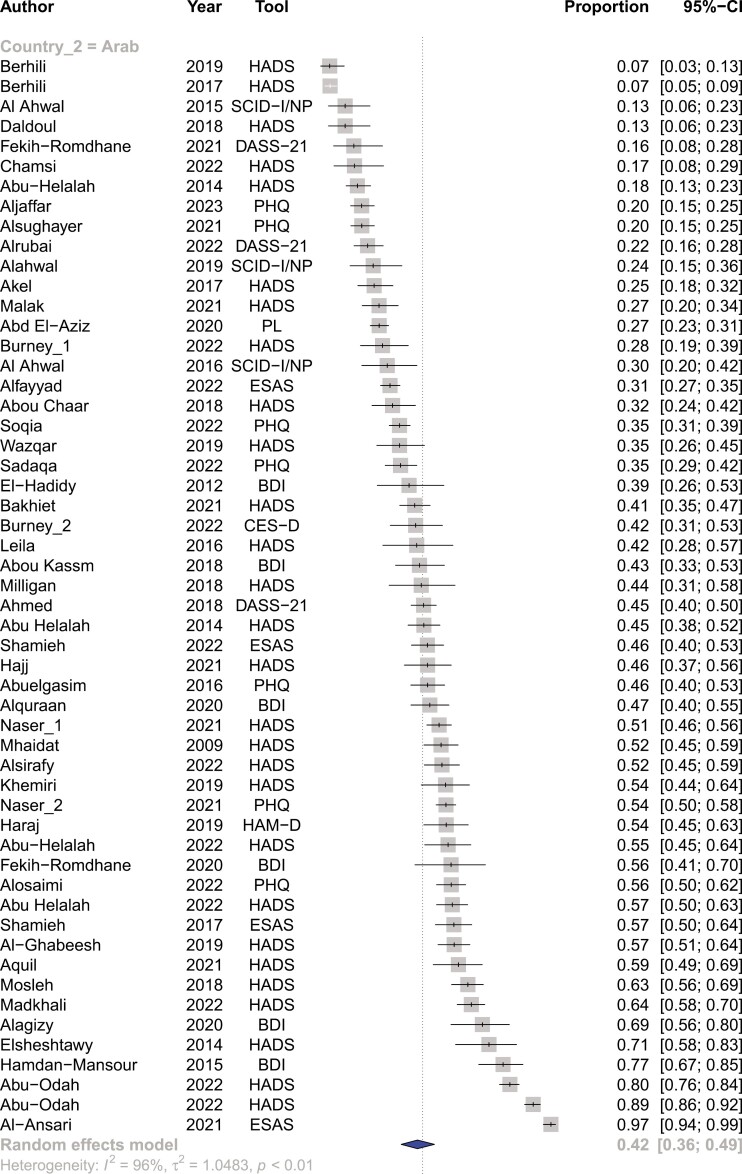

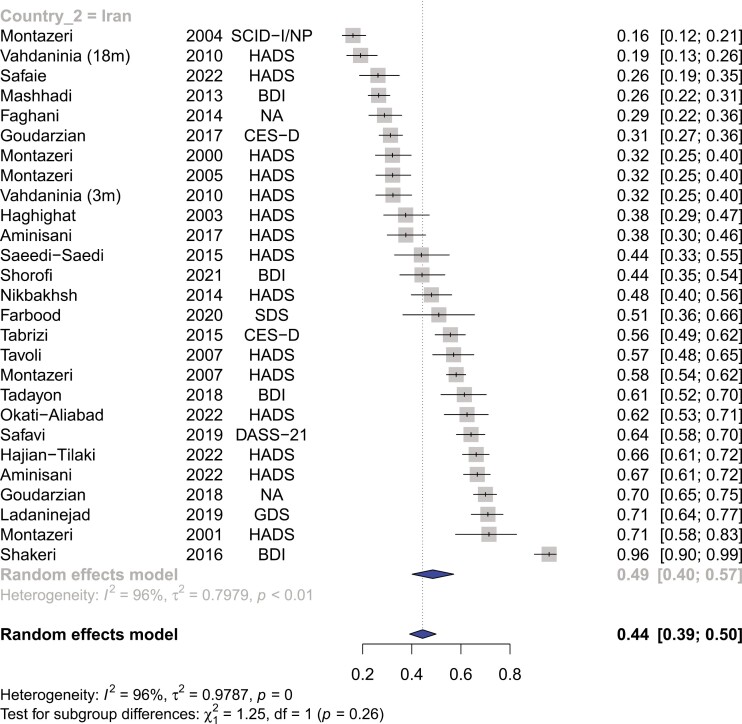
Pooled prevalence of depression among patients with cancer within Persian-speaking vs Arabic-speaking countries.

Among the included articles, the prevalence of depression was 44% (95%CI, 39%-50%) for patients with cancer residing within the MENA region (Refer to Figure 3). There were significant differences in the prevalence of depression among different countries ranging from 73% (95%CI, 42%-91%) in Palestine to 23% (95%CI, 7%-56%) in Morocco (Refer to Supplementary Figure S1). The prevalence of depression between Persian-Farsi speaking countries and Arabic-speaking countries was not significantly different (42% vs 49%, respectively) (refer to Figure 4). The prevalence of depression was significantly different among the various tools used to measure depression, particularly between DSM-V guided structured interviews vs questionnaire-based modalities such as HADS (refer to Supplementary Figure S2).

**Figure 5.**
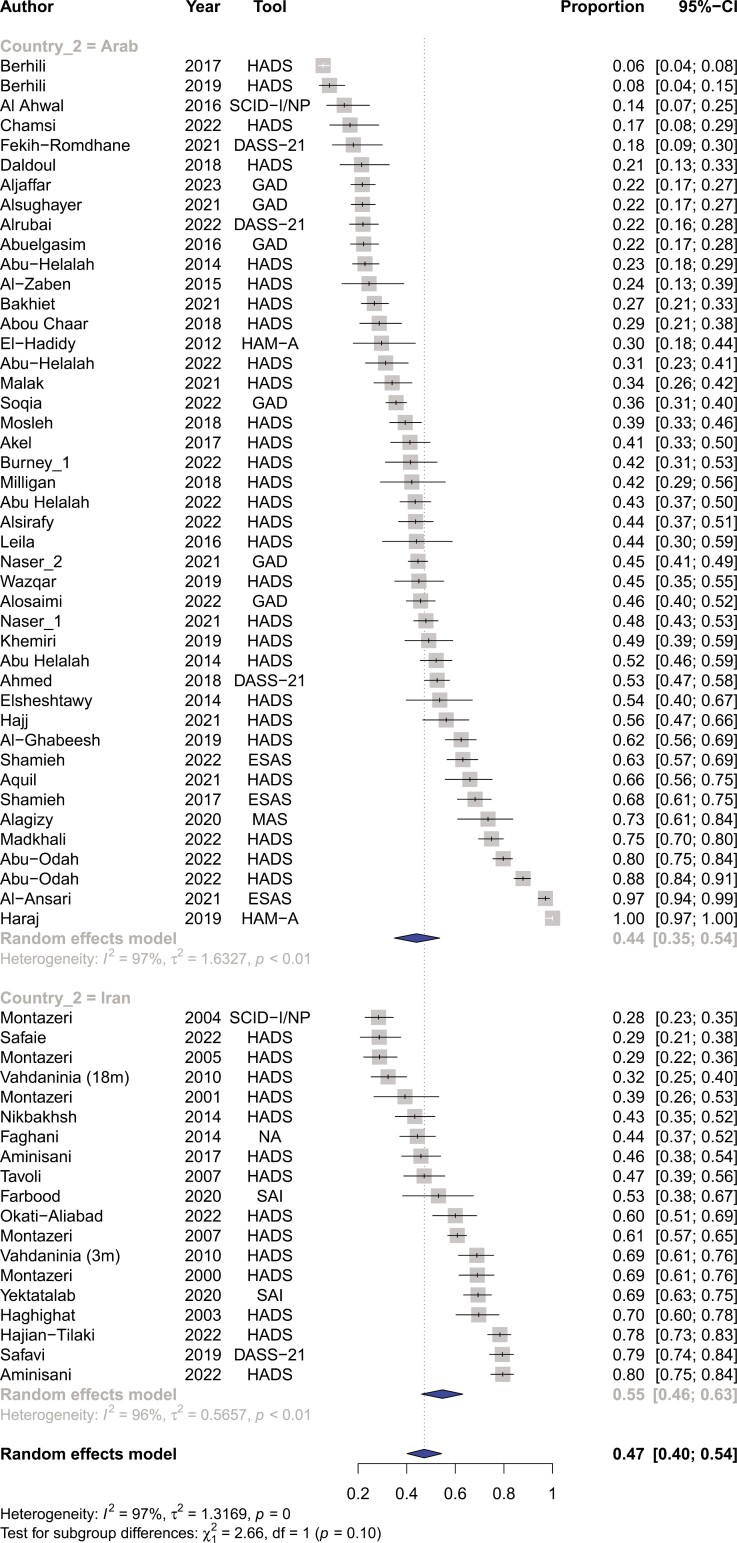
Pooled prevalence of anxiety among patients with cancer in the MENA region.

**Figure 6.**
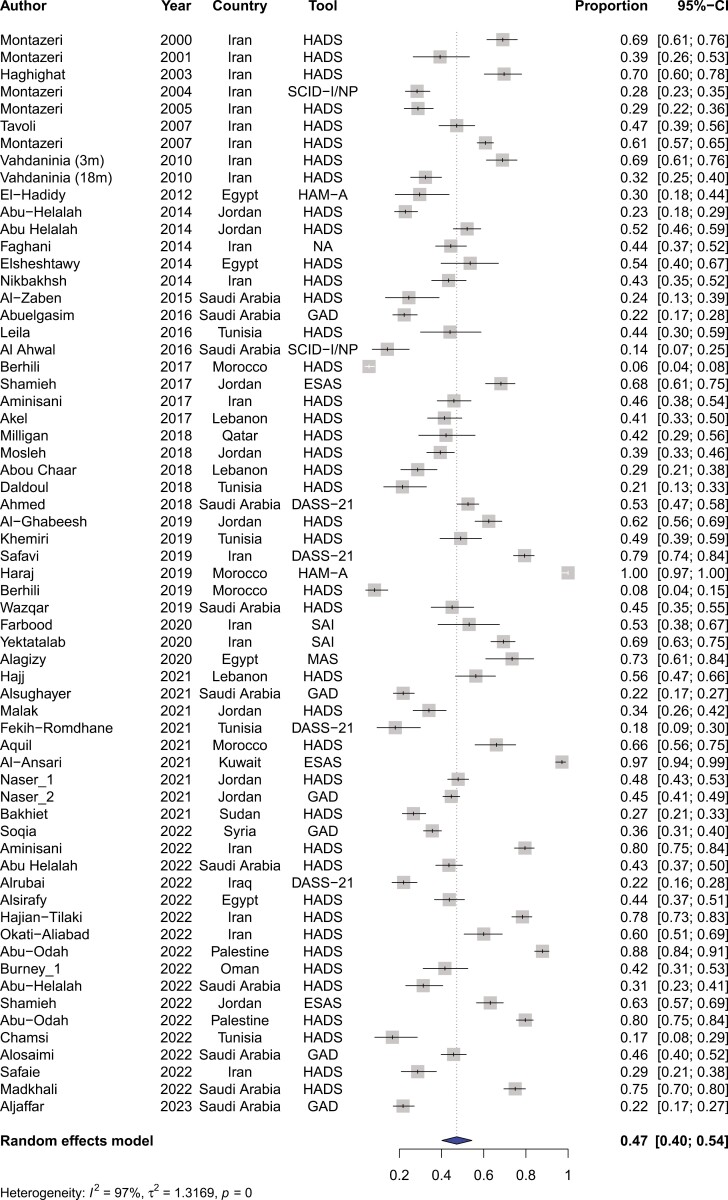
Pooled prevalence of anxiety among patients with cancer within Persian-speaking versus Arabic-speaking countries.

Across our pooled studies, the prevalence of anxiety among patients with cancer within the MENA region was 47% (95%CI, 40%-54%) (refer to Figure 5). The rates of anxiety prevalence were significantly different across MENA countries ranging from 64% (95%CI, 3%-99%) in Morocco to 28% (95%CI, 18%-42%) in Tunisia (refer to Supplementary Figure S3). Similar to depression, the prevalence of anxiety was not statistically different among Arabic-speaking and Persian-speaking countries (refer to Figure 6). Moreover, differences in the prevalence of anxiety among tools were significantly different, being the highest for the ESAS tool (83%; CI, 51%-96%) and lowest for the GAD (31%; CI, 23%-40%) (refer to Supplementary Figure S4).

**Figure 7.**
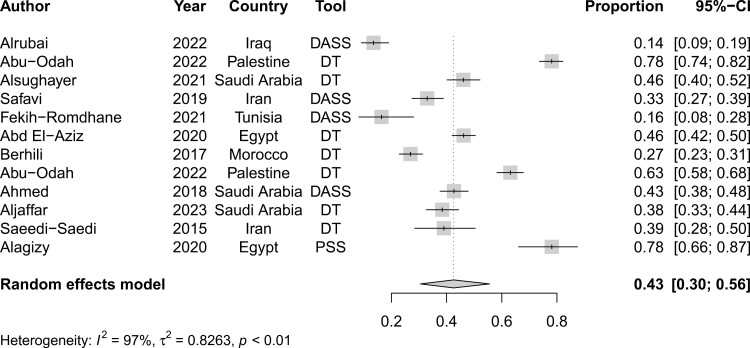
Pooled prevalence of distress among patients with cancer in the MENA region.

Among the 12 studies reporting on distress among patients with cancer in the MENA region, its prevalence was 43% (95%CI, 30%-56%) (refer to Figure 7).

### Psychiatric outcomes among patients with breast cancer

Depression was measured in 34 out of 36 studies conducted exclusively on patients with breast cancer. The prevalence of depression among this subgroup was 42% (95%CI, 34%-51%) (refer to Supplementary Figure S5). When stratified per country, Egypt had the highest prevalence of depression (60%; 95%CI, 43%-76%) compared to Tunisia (29%; 95%CI, 14%-50%) (refer to Supplementary Figure S6). There were no significant differences in the prevalence of depression among Arabic-speaking vs Persian-Farsi speaking patients with breast cancer (37% vs. 49%, respectively) (refer to Supplementary Figure S7). However, there were significant differences per measurement tool (refer to Supplementary Figure S8).

Across 27 studies reporting anxiety among patients with breast cancer, the pooled prevalence was 44% (CI, 35%-53%) (refer to Supplementary Figure S9) ranging from 59% (95%CI, 53%-65%) in Jordan to 26% (95%CI, 16%-41%) in Tunisia (refer to Supplementary Figure S10). Furthermore, it appears that Arabic-speaking patients have lower anxiety than their Persian-speaking counterparts (37% vs. 55%, respectively) (refer to Supplementary Figure S11). Finally, the prevalence of distress among patients with breast cancer was 39% (95%CI, 18%-64%).

### Meta regression

The prevalence of depression was neither impacted by publication year (*P* = .374) nor age (.091). Similarly, the prevalence of anxiety was not significantly affected by year of publication (*P* = .627) or age (*P* = .546).

### Heterogeneity among included studies

Heterogeneity was significantly high at 96% across studies reporting on depression (*P* < .001). When stratified by country, the highest level of heterogeneity was found for Palestine and Morocco at 99% and 98%, respectively. On the other hand, Lebanon and Tunisia had the lowest amount of heterogeneity at 82% and 91%, respectively. When stratified by tool, the highest levels of heterogeneity were found for the ESAS tool (*I*^2^ = 98%), followed by the DASS-21 (*I*^2^ = 97%), and the PHQ (*I*^2^ = 97%). In contrast, the lowest level of heterogeneity was observed in the SCID-I/NP method (*I*^2^ = 69%).

Likewise, there was significant heterogeneity at 96% among studies reporting on anxiety (*P* < .001). When stratified by country, Morocco, Iran, and Saudi Arabia had the highest rates of heterogeneity at 98%, 96%, and 96%, respectively. On the other hand, Tunisia (*I*^2^ = 87%), Lebanon (*I*^2^ = 88%), and Egypt (*I*^2^ = 88%) had the lowest rates of heterogeneity. Interestingly, heterogeneity was above 95% irrespective of used measurement tool. Finally, there was significant heterogeneity among the 12 studies reporting on distress at an *I*^2^ of 97% (*P* < .001).

## Discussion

To the best of the authors’ knowledge, this is the first meta-analysis to investigate the epidemiology of psychiatric diseases—namely anxiety, depression, and distress—among patients with cancer within the MENA region. In short, the overall pooled prevalence rates for depression, anxiety, and distress were 44%, 47%, and 43%, respectively. The prevalence rates for depression and anxiety were significantly different across MENA nations and measurement tools. Similar findings were replicated across studies reporting exclusively on patients with breast cancer in the MENA region.

Across the relevant literature, the overall prevalence rates for depression and anxiety exhibited noticeable variability. Ayubi et al demonstrated a pooled prevalence of depression and anxiety of 37% and 38%, respectively, among an international cohort of patients with cancer during the coronavirus disease 2019 (COVID-19) pandemic.^37^ Aryankhesal *et al* showed a pooled prevalence of depression of 35% among Iranian patients with cancer.^38^ Both of the aforementioned rates are comparable to the global rate of depression, which currently stands at 32·2% as of 2019.^39^ Interestingly, older reports from the early 2010s demonstrated significantly lower rates of depression and anxiety ranging from 10·8% to 16·0%.^40,41^ The phenomenon of increasing rates of psychological distress among patients with cancer across the past century could be attributed to the increased survivability of patients with cancer; an effect that is mediated by the advent of various forms of therapeutic options (eg, chemotherapy, neo/adjuvant therapy, immunotherapy) which are in of themselves associated with higher rates of psychiatric burden.^42^ The aforementioned is further supported by the observed links between psychological symptoms of cancer and treatment-related outcomes such as level of morbidity, disability, chronic pain, and experienced side effect profile.^43,44^

In addition to the impact generated by treatment method, the prevalence of psychiatric burden among patients with cancer is also influenced by a myriad of other factors including status of disease, time from cancer diagnosis, method of psychiatric burden measurement, and most interestingly, type of cancer. The prevalence of depression among patients with gastric and breast cancer revealed a pooled rate of 37·0% and 41·9%, respectively.^45^ On the other hand, the prevalence of depression for lung cancer, prostate cancer, and ovarian cancer is 3·0%, 17·3%, and 25·3%, respectively.^46-48^ The above mentioned can be explained by different clinical and social profiles exhibited by different cancers, which pertain to invasiveness and severity of treatment, access to proper healthcare, and societal stigma.

Juxtaposed against the previosly mentioned literature, the rates of psychiatric burden among patients with cancer in the MENA region are concerning. It appears that the psychiatric burden is amplified by economic, social, and cultural differences within the region.^49^ Patients with cancer within low-to-middle-income countries, which comprise the majority of MENA nations, are limited in terms of disposal income for cancer care whether it be screening, treatment, or follow-up.^38^ The scarcity of resources contributes to lower awareness of disease, poor treatment and follow-up, absence of screening programs, and late diagnosis of disease^50^; all of which are associated with significant psychological impact on patients and their caregivers. Other concerning sociocultural factors include the stigma associated with cancer, the use of complementary or alternative therapy (eg, faith healing), coping strategies, and social structures.^19^ For example, social and family support is observed as a mediator of psychiatric burden among patients with cancer.^51,52^ Thus, it is not presumptuous to assume the unique economic, social, and cultural profiles characterizing the MENA region might also be the reason behind the variability of experienced psychiatric burden across its different nations.

One extra factor that is fairly rampant within the MENA region is the presence of armed conflicts and political instability in some of its sectors. The literature has consistently observed the intertwined relationship between war and the burden of cancer.^53^ The rising incidence of cancer and the deterioration of its care has been documented in Iraq, Ukraine, and Palestine to name a few.^54-56^ Such burden is also amplified when other natural phenomena are in play, such as during the COVID-19 pandemic, which significantly hit patients residing or fleeing areas of conflict.^8^ Challenges to oncologists, entire healthcare systems, patients, and the supply chains of medications were implied in the dysfunctional processes of cancer diagnosis and treatment.^57^ From a psychological perspective, the association between war and immense mental health complications is well documented. Mental health challenges (eg, depression) are more prevalent among populations affected by war; a finding that was observed in Syria, Afghanistan, Palestine, and Ukraine among others.^54^ The psychological burden of these vulnerable populations is exacerbated by avoidant coping mechanisms to intense trauma, which could result in post-traumatic stress disorder and other unhealthy complications associated with certain coping strategies.^58-60^ Therefore, the impact of armed conflicts, while covert in discussions of MENA oncology care, is a key element in understanding and developing improved care strategies and policies.

### The psychology of cancer

The link between psychiatric burden and cancer is bidirectional and multifaceted. The literature and the existence of “psycho-oncology” have already established that the mere diagnosis of cancer comes with a significant psychiatric burden. However, it appears that such a burden is associated with the development and progress of cancer at the molecular level.^61,62^ Moreover, psychiatric diseases have been observed to impact disease-related behaviors (eg, adherence to treatment) and promote poor general health practices (eg, inactivity).^61,63^

Psychiatric burdens within the context of unhealthy lifestyle adjustments were observed to impact treatment compliance.^64^ Studies performed on various populations of patients with cancer including but not limited to prostate cancer, breast cancer, and pancreatic cancer, demonstrated lower compliance in those with either anxiety or depression.^65-68^ DiMatteo *et al*. estimated that, generally, depressed patients are 3 times more likely to be non-compliant with treatment recommendations.^69^ Thus, it is only expected that psychiatric illnesses, such as depression, were shown to have a negative prognostic value in predicting both cancer recurrence and survival.^70,71^

The field of psycho-oncology is a relatively new discipline that is yet to be up-taken by cancer institutions worldwide. The International Psycho-Oncology Society (IPOS) has issued an International Standard of Quality of Cancer Care which called for the integration of the psychosocial domains as core aspects of holistic cancer care, which labeled it as the “sixth vital sign.”^72^ The expansion of psycho-oncology led to the adoption of distress screening and the development of a multitude of psycho-oncologic interventions^72,73^; all of which are associated with improved patients’ psychiatric outcomes at both the behavioral^74-77^ and molecular levels.^78-80^

### Challenges to psycho-oncology in the MENA region

Paucity of research is the most pertinent challenge impeding the initiation, development, and acceptance of any psycho-oncology program within the MENA region. For nearly an entire decade, literature describing the epidemiological characteristics of depression and anxiety was primarily conducted in Iran, only for it to accumulate pace in the last 5 years along certain MENA nations such as Jordan and Saudi Arabia. Huge nations, in terms of both size and population, such as Iraq, Sudan, and Syria had only one study each documenting the rates of psychiatric outcomes among their patients with cancer. The dearth of research is best exemplified in the lack of validation studies for tools for measuring psychiatric outcomes associated with cancer across many MENA nations.

The other barriers to psycho-oncology are systematic (ie, healthcare-related) and sociocultural barriers. Systematic barriers mainly pertain to resource scarcity.^18^ Considering such a limitation, cancer care institutions within the MENA region are primarily focused on the treatment dimension of cancer care delivery.^7^ Thus, psycho-social and palliative care services may not be available or extremely expensive. From a patient-centered perspective, resource limitations influence both adherence and follow-up.^19^ Patients with low levels of income are more likely to ignore psychotherapy sessions, psychiatric referrals, and mental health medications. In fact, due to a number of financial and geographic barriers, patients might not be able to follow up on their clinical disease, let alone the progression of their disease-augmenting psychiatric illness. This was documented in the Lebanese experience with psycho-oncology.^19^

On the other hand, sociocultural barriers exist at both the individual and community levels. For instance, seeking treatment for already existing psychological needs is a taboo in Arab societies.^81^ Additionally, due to the collectivist culture of Arab social units, the decision to pursue psycho-oncological care may not always reside with the affected patient, but rather with caregivers and intimate social circles.^17,18,81^ This unique characteristic of the Arab culture may enhance or suppress participation in psycho-oncology programs.

A number of psycho-oncology programs and groups have been described within the MENA region.^18,81^ Those include the King Hussein Cancer Center, Societe Tunisienne de PsychoOncologie, the Children’s Cancer Hospital Egypt 57357, El Qabbary Specialist Hospital Clinical Oncology and Palliative Care Center, and the American University of Beirut Medical Center. While limited in number, potential trials on the effectiveness of such programs are yet to grace the literature. In fact, aside from mere descriptions of provided psycho-oncology services, details with regard to types, coverage, duration, and impact of such services are not illuminated upon.

### The psychometric status of MENA region

In our analysis, we demonstrated that different measurement tools provided different estimates of psychiatric burden; an observation consistent with other similar meta-analyses.^37,45^ The literature notes that certain tools such as the ESAS and GAD/PHQ showed higher prevalence values of anxiety and depression when compared with HADS.^37,82^ Similarly, when compared to SCID, it has been shown that the PHQ-9 at a cutoff point of 10 significantly overestimates the prevalence of depression.^83^ This variance, complemented by inconsistent definitions of psychiatric disorders and diverse cutoff points for categorization, implies that an accurate estimation of psychiatric morbidity may not always be feasible, as a function of methodological heterogeneity. Enough evidence on the comparative efficacy of different measurement tools is yet to be established; however, some evidence suggests that the HADS tool is the most optimal for identifying anxiety and depression.^84-87^ This is mostly due to the fact that the tool is associated with better compliance by patients, shorter time to completion, good correlations with clinical features, and appropriate psychometric settings.

Within the context of psycho-oncology, the research landscape within the MENA region is mainly descriptive with little movement toward interventional research. With the exception of Iran, the Arab world has barely any large-scale publications reporting on psycho-oncological interventions. Chambers et al note that for psycho-oncological interventional studies to be conducted and of which results are to be disseminated, longitudinal descriptive studies using appropriate measurement tools must be achieved.^88^ Thus, it is questionable to provide inferences on findings relevant to a certain culture using tools developed for the contextual needs of another.^89^

Considering that questionnaire-based measurement tools are cornerstone to psycho-oncology research, the stability of their psychometric properties is crucial in providing accurate estimates. Throughout the studies aggregated within this review, psychiatric burden was measured by tools that were validated and translated a century ago. This raises points of skepticism regarding the psychometric appropriateness of such tools considering the wide range of dialects of the Arabic language and different perceptions of psychiatric illness. The issue of cultural adaptability also extends to the reliability of cutoff scores as each culture/region might be associated with different thresholds for different psychiatric illnesses.

Another issue pertaining to psychometrics lies in the lack of tools for measuring psychiatric burden for individual cancers. It may be naïve to assume that the link between cancer and psychological burden is similar across different forms, stages, and severities of cancer. Therefore, dedicated tools for measuring psycho-social outcomes should be developed for the most commonly prevalent cancers within the MENA region.^42^

### Proposed recommendations

Compared to Iran, the psycho-oncology literature in the Arab world is lacking. In fact, many Arab nations are barely represented in even reporting the prevalence rates of psychiatric burden. Moreover, with the exception of breast and colorectal cancers, the psychiatric impact of individual cancers cannot be reliably estimated. The literature also suffers from questionable methodological errors mainly pertaining to questionnaire and cutoff validation or recruited sample sizes; both of which may impact the generalizability of its findings. The lack of culturally sensitive tools, insufficient funding, and stigma are often cited as the most common hurdles to mental health research output and advancement within the Arab world.^90^ Therefore, it is crucial for future research to attempt to illustrate the burden of psycho-oncology using appropriate tools and across representative samples of moderate size throughout the MENA nations.

Cultural beliefs and myths may amplify stigmatizing behaviors across both cancer and psychiatric illness. Although in most cases, the major barrier cited hindering the help-seeking behavior is the lack of perceived need rather than stigma. Nonetheless, it is crucial to implement awareness campaigns that target both concepts to reveal the importance of psycho-oncology services through emphasizing ideas of autonomy, early diagnosis, and all-encompassing treatment modalities in improving overall quality of life and alleviating the stigmatizing connotations adhered to them.^91^ Such campaigns must be culturally sensitive as to accommodate patients’ cultural heritage, values, morals, and family structure. The impact of cancer on the latter justifies the extension of such efforts to caregivers.

Most importantly, steps to remedy lack of funds and inappropriate allocation of resources must be considered to overcome barriers to advancing holistic cancer care. Securing funding from local and international bodies, investing in telemedicine technology, upgrading infrastructure, and updating treatment policies are some of the steps that must be considered when attempting to improve the spectrum of cancer care, particularly psycho-oncology, among institutions.^18,92^

Psycho-oncological therapies have demonstrated varying degrees of success in alleviating the burden of psychiatric burden.^93^ Concerned healthcare systems with ample resources should strive to integrate such therapies within their cancer care programs. Moreover, these concerned bodies should explore the impact of such programs on proxy markers of treatment success such as uptake, sustenance, and compliance. However, the literature also shows that the therapeutic alliance between patient and physician is pertinent to positive treatment outcomes ranging from compliance to quality of life.^94^ Thus, it is important to provide healthcare personnel with the appropriate training and skills to be able to contain the psycho-oncologic complications of patients.

### Limitations

A number of limitations are cited within this review. First, the quality of pooled evidence may not be indicative of its representing populations due to the dominant cross-sectional nature of included studies. Second, only articles published in the English language were included which could have led to the exclusion of Persian-, French-, or Arabic-only studies documenting our outcomes of interest. Third, not all MENA nations were represented by a sufficient number of well-powered studies. This could skew our findings toward certain geographic areas and limit their generalizability. Fourth, the impact of cancer characteristics on depression was not evaluated. Due to the limited availability of information, details on stage, treatment, and other clinical factors were not considered. Fifth, despite conducting sensitivity analysis, subgroup analysis, and meta-regression, the heterogeneity among studies was extremely high. Such is expected due to the limited quality of study designs and extreme variance in implementing measurement tools. Sixth, due to the lack of normative data for many of the targeted MENA populations, other measures of psychiatric burden including but not limited to quality of life, sleep quality, pain, and fatigue were not accounted for. Seventh, of the included 83 studies, 21 studies had a total sample size equal to or less than 100 participants, which could have introduced a small-study effect into our pooled estimates. Finally, only 3 databases were examined for relevant literature which could have introduced the risk of selection bias.

## Conclusion

In summary, our review demonstrated that the psychological burden among patients with cancer residing within the MENA region is significantly high. Moreover, this burden is heterogeneous across different MENA nations and even different measurement tools. Due to the impact of the psychological burden of cancer on patients’ mortality, morbidity, and overall quality of life, policymakers should seriously consider the adoption or creation of appropriate psycho-oncology services. Also, future research endeavors should attempt to create and validate psycho-oncologic measurement tools that are appropriate to the culture of targeted patients and their clinical characteristics, particularly type of cancer. Finally, our results provided justification for the implementation of routine screening, assessment, and management of psychological disorders among patients with cancer.

## Supplementary material

Supplementary material is available at *The Oncologist* online.

oyae193_suppl_Supplementary_Material

## Data Availability

All data/datasets associated with this project will be provided at a reasonable request from the corresponding author.
