# Supplementary material for: Assessment of psycho-oncology in the Middle East and North Africa region: a systematic review and meta-analysis
Source: Oncologist. 2024 Aug 13;29(11):e1452–69. doi: 10.1093/oncolo/oyae193 (PMC11546821; doi:10.1093/oncolo/oyae193)

Assessment of psycho-oncology in the MENA region: A systematic review and meta-analysis

Supplementary Material

Search query

Variable	Keywords
Cancer	“cancer” (and synonyms/MeSH terms). Synonyms include: neoplasms, metastasis, malignancies, etc.
Psycho-oncology outcomes	“well-being”, “coping”, “anxiety”, “depression”, “pain”, “fatigue”, “quality of life”, “distress” among others (and synonyms/MeSH terms).
MENA populations	The names of countries included within the MENA region, as defined by the Royal College of Pathology (RCPath), along with the adjectives describing nationality, will be used to perform the search.

The final query for the above-mentioned objective is as follows: ((Distress OR depression OR anxiety OR coping OR emotional adjustment OR social adjustment OR psychological OR psychosocial)) AND (Cancer OR "Neoplasms"[Mesh] OR metastasis) AND ("Algeria" OR "Bahrain" OR "Djibouti" OR "Egypt" OR "Palestine" OR "Iran" OR "Iraq" OR "Jordan" OR "Kuwait" OR "Lebanon" OR "Libya" OR "Morocco" OR "Oman" OR "Qatar" OR "Saudi Arabia" OR "Somalia" OR "Sudan" OR "Syria" OR "Tunisia" OR "United Arab Emirates" OR "Yemen")).

Supplementary figures

Figure 1: Prevalence of depression stratified by country

Figure 2: Prevalence of depression stratified by measurement tool

Figure 3: Prevalence of anxiety stratified by country

Figure 4: Prevalence of anxiety stratified by measurement tool

Figure 5: Prevalence of depression among patients with breast cancer in the MENA region

Figure 6: Prevalence of depression among patients with breast cancer in the MENA region stratified by country

Figure 7: Pooled prevalence of depression among patients with breast cancer within Persian-speaking versus Arabic-speaking countries

Figure 8: Prevalence of depression among patients with breast cancer in the MENA region stratified by measurement tool

Figure 9: Prevalence of anxiety among patients with breast cancer in the MENA region

Figure 10: Prevalence of anxiety among patients with breast cancer in the MENA region stratified by country

Figure 11: Pooled prevalence of anxiety among patients with breast cancer within Persian-speaking versus Arabic-speaking countries

Figure 12: Prevalence of anxiety among patients with breast cancer in the MENA region stratified by measurement tool

Figure 13: Funnel plot for articles reporting on depression

Figure 14: Funnel plot for articles reporting on anxiety

Supplementary figure 1

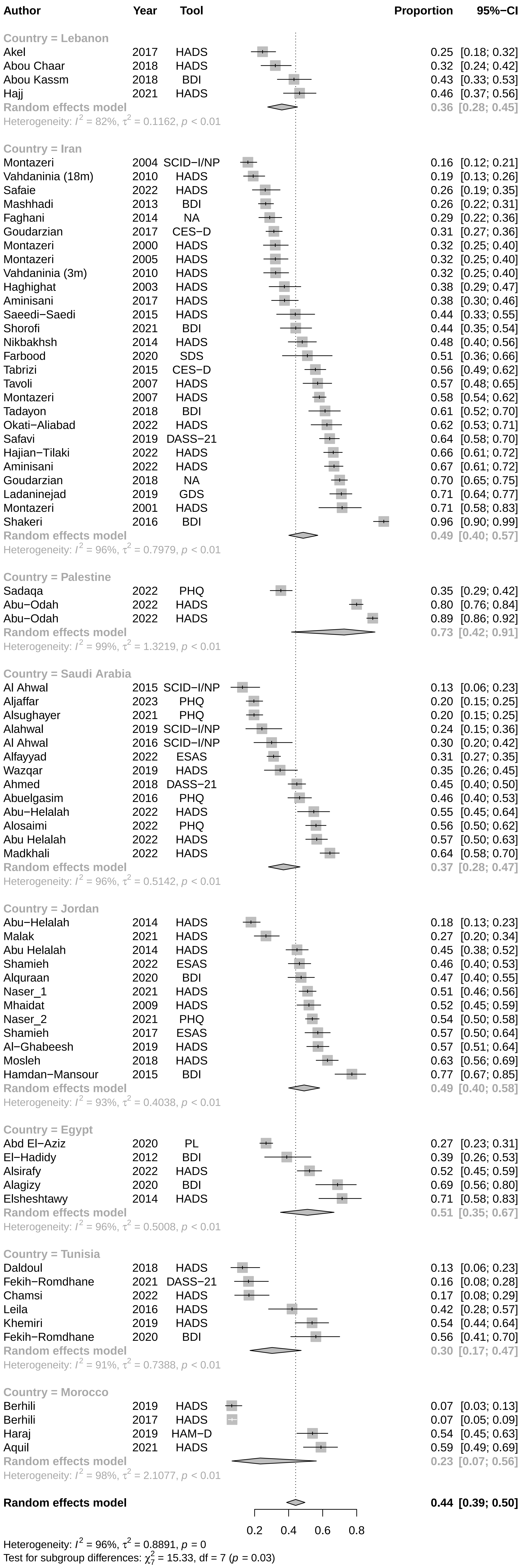

Supplementary figure 2

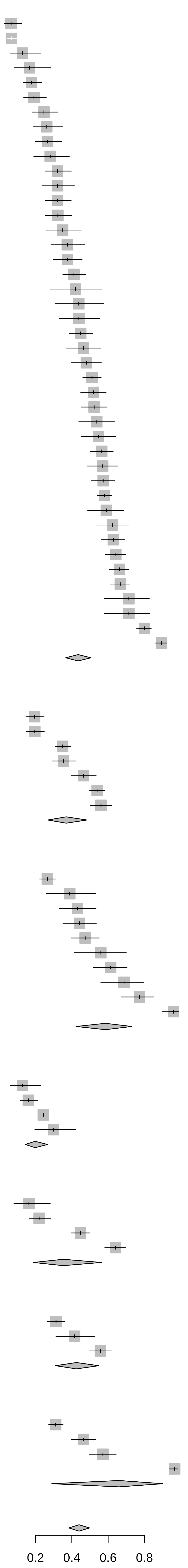

Author	Year	Country	Proportion	95%−CI
**Tool_Depression = HADS**				
Berhili	2019	Morocco	0.07	[0.03; 0.13]
Berhili	2017	Morocco	0.07	[0.05; 0.09]
Daldoul	2018	Tunisia	0.13	[0.06; 0.23]
Chamsi	2022	Tunisia	0.17	[0.08; 0.29]
Abu−Helalah	2014	Jordan	0.18	[0.13; 0.23]
Vahdaninia (18m)	2010	Iran	0.19	[0.13; 0.26]
Akel	2017	Lebanon	0.25	[0.18; 0.32]
Safaie	2022	Iran	0.26	[0.19; 0.35]
Malak	2021	Jordan	0.27	[0.20; 0.34]
Burney_1	2022	Oman	0.28	[0.19; 0.39]
Montazeri	2000	Iran	0.32	[0.25; 0.40]
Abou Chaar	2018	Lebanon	0.32	[0.24; 0.42]
Montazeri	2005	Iran	0.32	[0.25; 0.40]
Vahdaninia (3m)	2010	Iran	0.32	[0.25; 0.40]
Wazqar	2019	Saudi Arabia	0.35	[0.26; 0.45]
Haghighat	2003	Iran	0.38	[0.29; 0.47]
Aminisani	2017	Iran	0.38	[0.30; 0.46]
Bakhiet	2021	Sudan	0.41	[0.35; 0.47]
Leila	2016	Tunisia	0.42	[0.28; 0.57]
Milligan	2018	Qatar	0.44	[0.31; 0.58]
Saeedi−Saedi	2015	Iran	0.44	[0.33; 0.55]
Abu Helalah	2014	Jordan	0.45	[0.38; 0.52]
Hajj	2021	Lebanon	0.46	[0.37; 0.56]
Nikbakhsh	2014	Iran	0.48	[0.40; 0.56]
Naser_1	2021	Jordan	0.51	[0.46; 0.56]
Mhaidat	2009	Jordan	0.52	[0.45; 0.59]
Alsirafy	2022	Egypt	0.52	[0.45; 0.59]
Khemiri	2019	Tunisia	0.54	[0.44; 0.64]
Abu−Helalah	2022	Saudi Arabia	0.55	[0.45; 0.64]
Abu Helalah	2022	Saudi Arabia	0.57	[0.50; 0.63]
Tavoli	2007	Iran	0.57	[0.48; 0.65]
Al−Ghabeesh	2019	Jordan	0.57	[0.51; 0.64]
Montazeri	2007	Iran	0.58	[0.54; 0.62]
Aquil	2021	Morocco	0.59	[0.49; 0.69]
Okati−Aliabad	2022	Iran	0.62	[0.53; 0.71]
Mosleh	2018	Jordan	0.63	[0.56; 0.69]
Madkhali	2022	Saudi Arabia	0.64	[0.58; 0.70]
Hajian−Tilaki	2022	Iran	0.66	[0.61; 0.72]
Aminisani	2022	Iran	0.67	[0.61; 0.72]
Montazeri	2001	Iran	0.71	[0.58; 0.83]
Elsheshtawy	2014	Egypt	0.71	[0.58; 0.83]
Abu−Odah	2022	Palestine	0.80	[0.76; 0.84]
Abu−Odah	2022	Palestine	0.89	[0.86; 0.92]
**Random effects model**			**0.43**	**[0.37; 0.51]**
Heterogeneity: $I^2 = 96\%$, $\tau^2 = 0.8737$, $p < 0.01$				
**Tool_Depression = PHQ**				
Aljaffar	2023	Saudi Arabia	0.20	[0.15; 0.25]
Alsughayer	2021	Saudi Arabia	0.20	[0.15; 0.25]
Soqia	2022	Syria	0.35	[0.31; 0.39]
Sadaqa	2022	Palestine	0.35	[0.29; 0.42]
Abuelgasim	2016	Saudi Arabia	0.46	[0.40; 0.53]
Naser_2	2021	Jordan	0.54	[0.50; 0.58]
Alosaimi	2022	Saudi Arabia	0.56	[0.50; 0.62]
**Random effects model**			**0.37**	**[0.27; 0.48]**
Heterogeneity: $I^2 = 97\%$, $\tau^2 = 0.3823$, $p < 0.01$				
**Tool_Depression = BDI**				
Mashhadi	2013	Iran	0.26	[0.22; 0.31]
El−Hadidy	2012	Egypt	0.39	[0.26; 0.53]
Abou Kassm	2018	Lebanon	0.43	[0.33; 0.53]
Shorofi	2021	Iran	0.44	[0.35; 0.54]
Alquraan	2020	Jordan	0.47	[0.40; 0.55]
Fekih−Romdhane	2020	Tunisia	0.56	[0.41; 0.70]
Tadayon	2018	Iran	0.61	[0.52; 0.70]
Alagizy	2020	Egypt	0.69	[0.56; 0.80]
Hamdan−Mansour	2015	Jordan	0.77	[0.67; 0.85]
Shakeri	2016	Iran	0.96	[0.90; 0.99]
**Random effects model**			**0.59**	**[0.42; 0.73]**
Heterogeneity: $I^2 = 94\%$, $\tau^2 = 1.0504$, $p < 0.01$				
**Tool_Depression = SCID−I/NP**				
Al Ahwal	2015	Saudi Arabia	0.13	[0.06; 0.23]
Montazeri	2004	Iran	0.16	[0.12; 0.21]
Alahwal	2019	Saudi Arabia	0.24	[0.15; 0.36]
Al Ahwal	2016	Saudi Arabia	0.30	[0.20; 0.42]
**Random effects model**			**0.20**	**[0.14; 0.27]**
Heterogeneity: $I^2 = 69\%$, $\tau^2 = 0.0877$, $p = 0.02$				
**Tool_Depression = DASS−21**				
Fekih−Romdhane	2021	Tunisia	0.16	[0.08; 0.28]
Alrubai	2022	Iraq	0.22	[0.16; 0.28]
Ahmed	2018	Saudi Arabia	0.45	[0.40; 0.50]
Safavi	2019	Iran	0.64	[0.58; 0.70]
**Random effects model**			**0.35**	**[0.19; 0.56]**
Heterogeneity: $I^2 = 97\%$, $\tau^2 = 0.7246$, $p < 0.01$				
**Tool_Depression = CES−D**				
Goudarzian	2017	Iran	0.31	[0.27; 0.36]
Burney_2	2022	Oman	0.42	[0.31; 0.53]
Tabrizi	2015	Iran	0.56	[0.49; 0.62]
**Random effects model**			**0.43**	**[0.31; 0.55]**
Heterogeneity: $I^2 = 95\%$, $\tau^2 = 0.1688$, $p < 0.01$				
**Tool_Depression = ESAS**				
Alfayyad	2022	Saudi Arabia	0.31	[0.27; 0.35]
Shamieh	2022	Jordan	0.46	[0.40; 0.53]
Shamieh	2017	Jordan	0.57	[0.50; 0.64]
Al−Ansari	2021	Kuwait	0.97	[0.94; 0.99]
**Random effects model**			**0.66**	**[0.29; 0.90]**
Heterogeneity: $I^2 = 98\%$, $\tau^2 = 2.4906$, $p < 0.01$				
**Random effects model**			**0.44**	**[0.38; 0.50]**

Heterogeneity: $I^2 = 96\%$, $\tau^2 = 1.0109$, $p = 0$
Test for subgroup differences: $\chi^2_6 = 32.52$, df = 6 ($p < 0.01$)

Supplementary figure 3

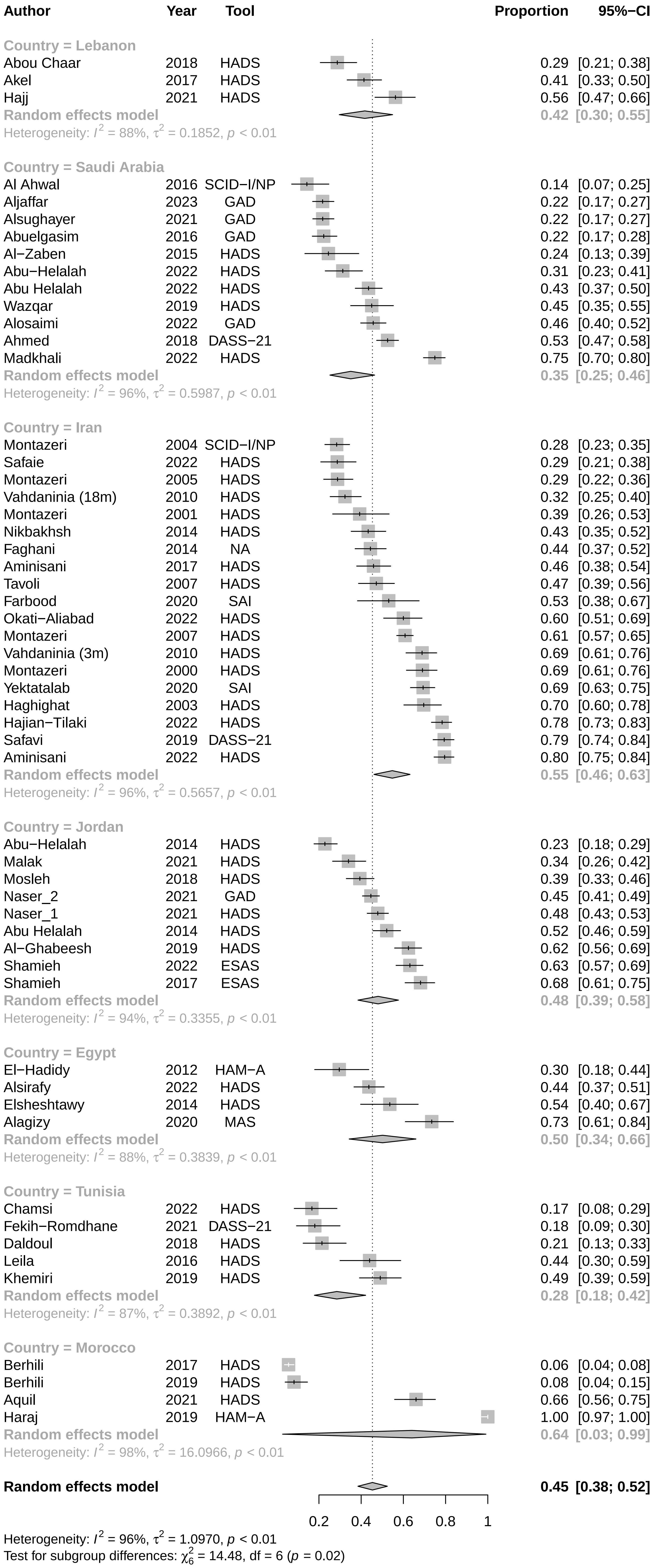

Supplementary figure 4

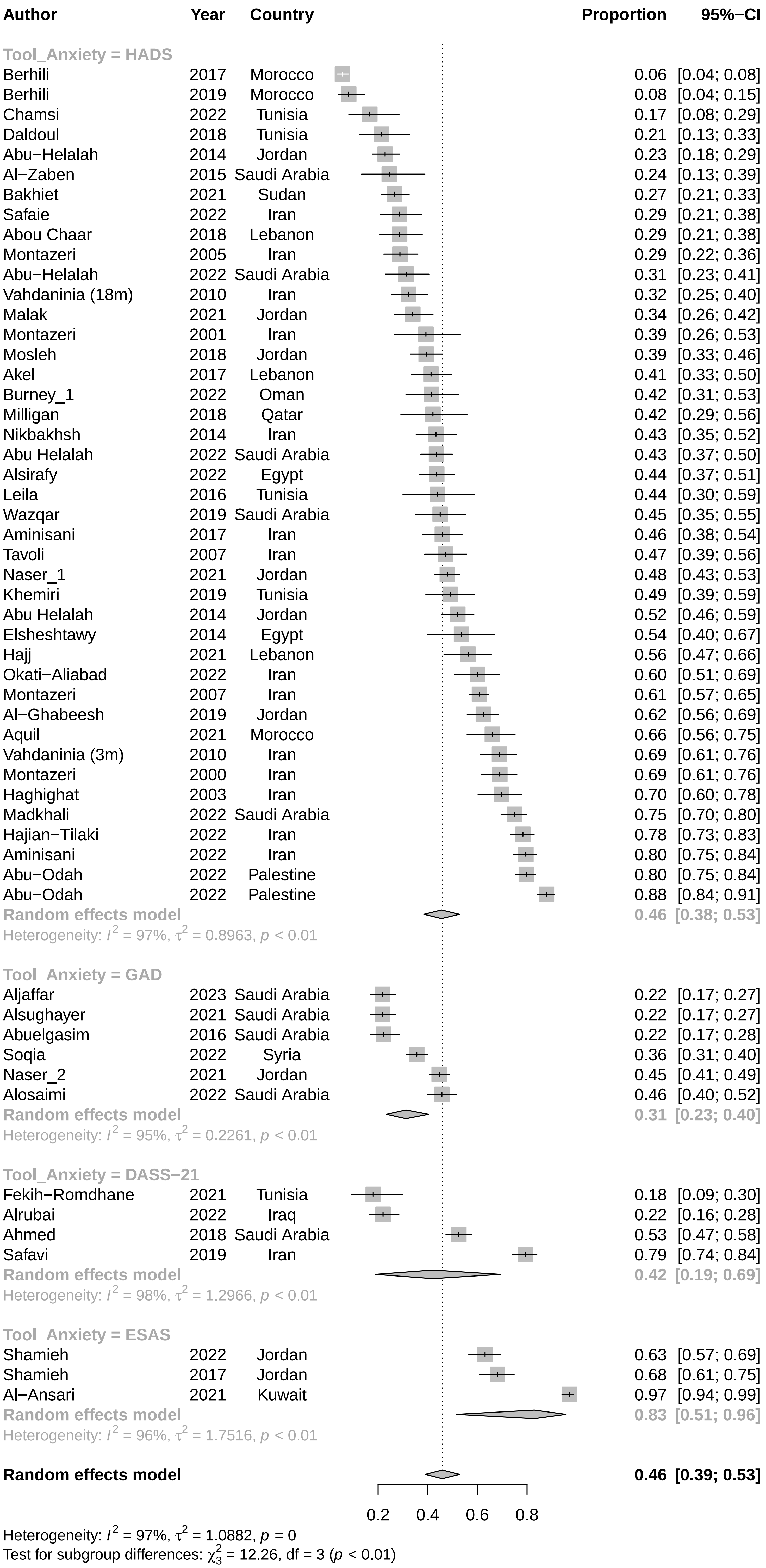

Supplementary figure 5

Author	Year	Country	Tool	Proportion	95%−CI
Montazeri	2000	Iran	HADS	0.32	[0.25; 0.40]
Montazeri	2001	Iran	HADS	0.71	[0.58; 0.83]
Haghighat	2003	Iran	HADS	0.38	[0.29; 0.47]
Montazeri	2004	Iran	SCID−I/NP	0.16	[0.12; 0.21]
Montazeri	2005	Iran	HADS	0.32	[0.25; 0.40]
Vahdaninia (3m)	2010	Iran	HADS	0.32	[0.25; 0.40]
Vahdaninia (18m)	2010	Iran	HADS	0.19	[0.13; 0.26]
El−Hadidy	2012	Egypt	BDI	0.39	[0.26; 0.53]
Abu Helalah	2014	Jordan	HADS	0.45	[0.38; 0.52]
Elsheshtawy	2014	Egypt	HADS	0.71	[0.58; 0.83]
Tabrizi	2015	Iran	CES−D	0.56	[0.49; 0.62]
Saeedi−Saedi	2015	Iran	HADS	0.44	[0.33; 0.55]
Shakeri	2016	Iran	BDI	0.96	[0.90; 0.99]
Leila	2016	Tunisia	HADS	0.42	[0.28; 0.57]
Berhili	2017	Morocco	HADS	0.07	[0.05; 0.09]
Akel	2017	Lebanon	HADS	0.25	[0.18; 0.32]
Abou Kassm	2018	Lebanon	BDI	0.43	[0.33; 0.53]
Tadayon	2018	Iran	BDI	0.61	[0.52; 0.70]
Daldoul	2018	Tunisia	HADS	0.13	[0.06; 0.23]
Al−Ghabeesh	2019	Jordan	HADS	0.57	[0.51; 0.64]
Berhili	2019	Morocco	HADS	0.07	[0.03; 0.13]
Farbood	2020	Iran	SDS	0.51	[0.36; 0.66]
Fekih−Romdhane	2020	Tunisia	BDI	0.56	[0.41; 0.70]
Alquraan	2020	Jordan	BDI	0.47	[0.40; 0.55]
Alagizy	2020	Egypt	BDI	0.69	[0.56; 0.80]
Hajj	2021	Lebanon	HADS	0.46	[0.37; 0.56]
Shorofi	2021	Iran	BDI	0.44	[0.35; 0.54]
Fekih−Romdhane	2021	Tunisia	DASS−21	0.16	[0.08; 0.28]
Soqia	2022	Syria	PHQ	0.35	[0.31; 0.39]
Sadaqa	2022	Palestine	PHQ	0.35	[0.29; 0.42]
Abu Helalah	2022	Saudi Arabia	HADS	0.57	[0.50; 0.63]
Hajian−Tilaki	2022	Iran	HADS	0.66	[0.61; 0.72]
Okati−Aliabad	2022	Iran	HADS	0.62	[0.53; 0.71]
Shamieh	2022	Jordan	ESAS	0.46	[0.40; 0.53]
**Random effects model**				**0.42**	**[0.34; 0.51]**

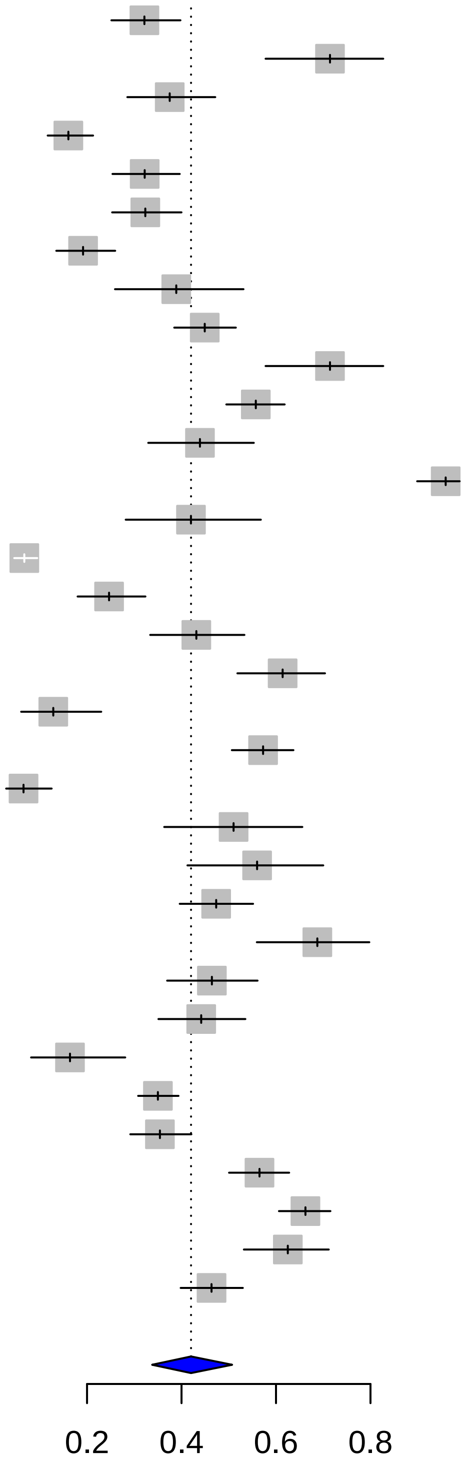

Heterogeneity: I^2 = 95%, τ^2 = 1.0385, p < 0.01

Supplementary figure 6

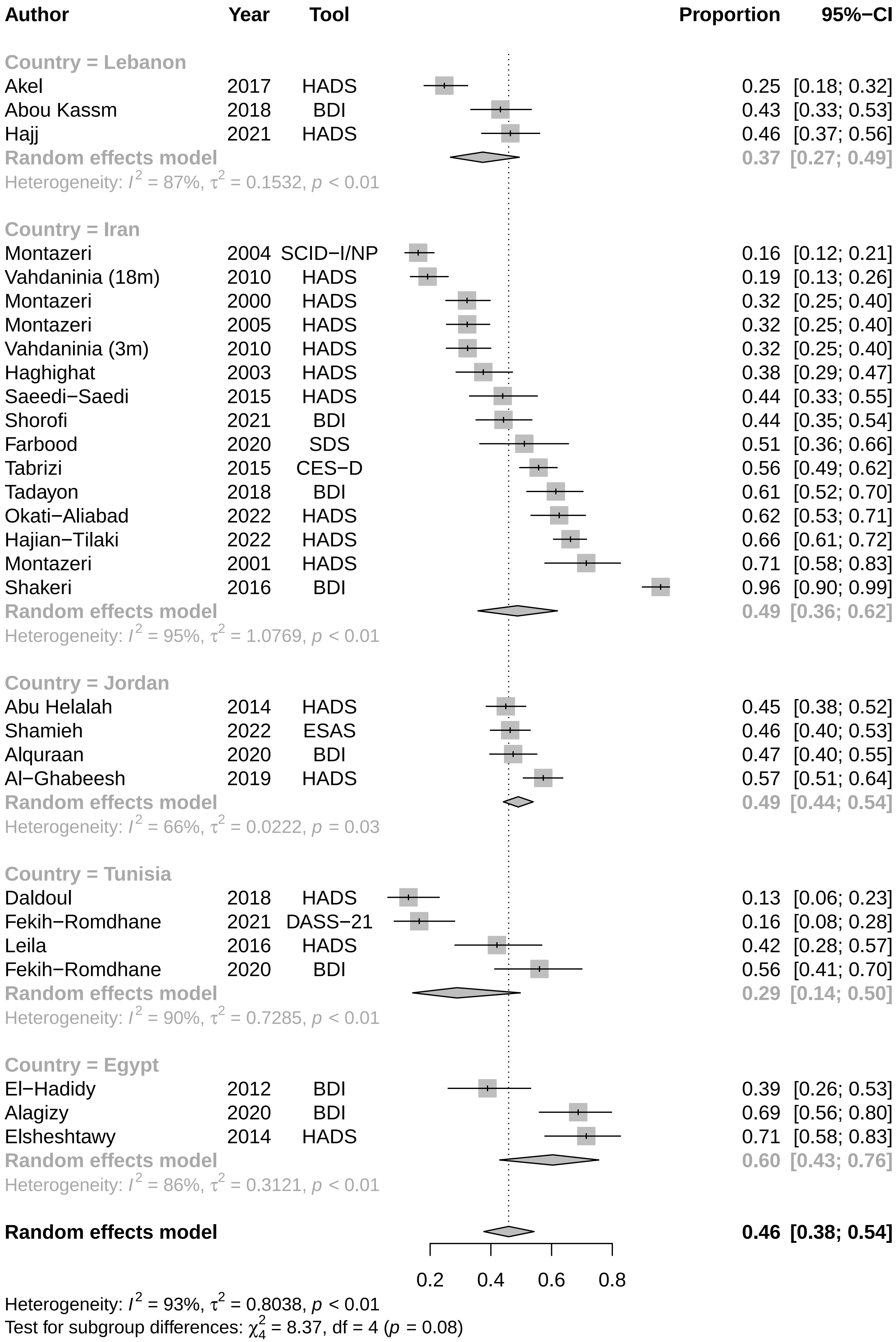

Supplementary figure 7

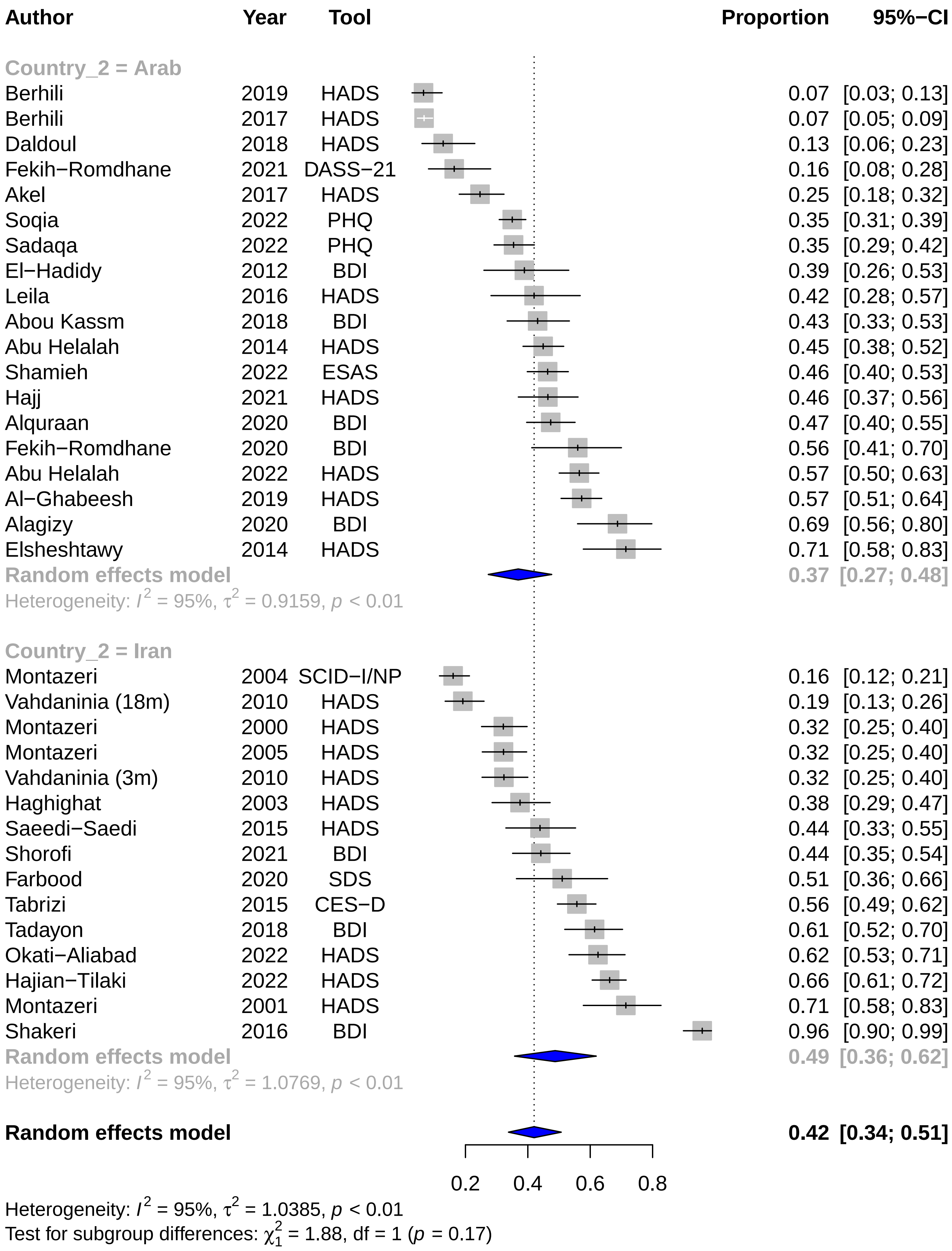

Supplementary figure 8

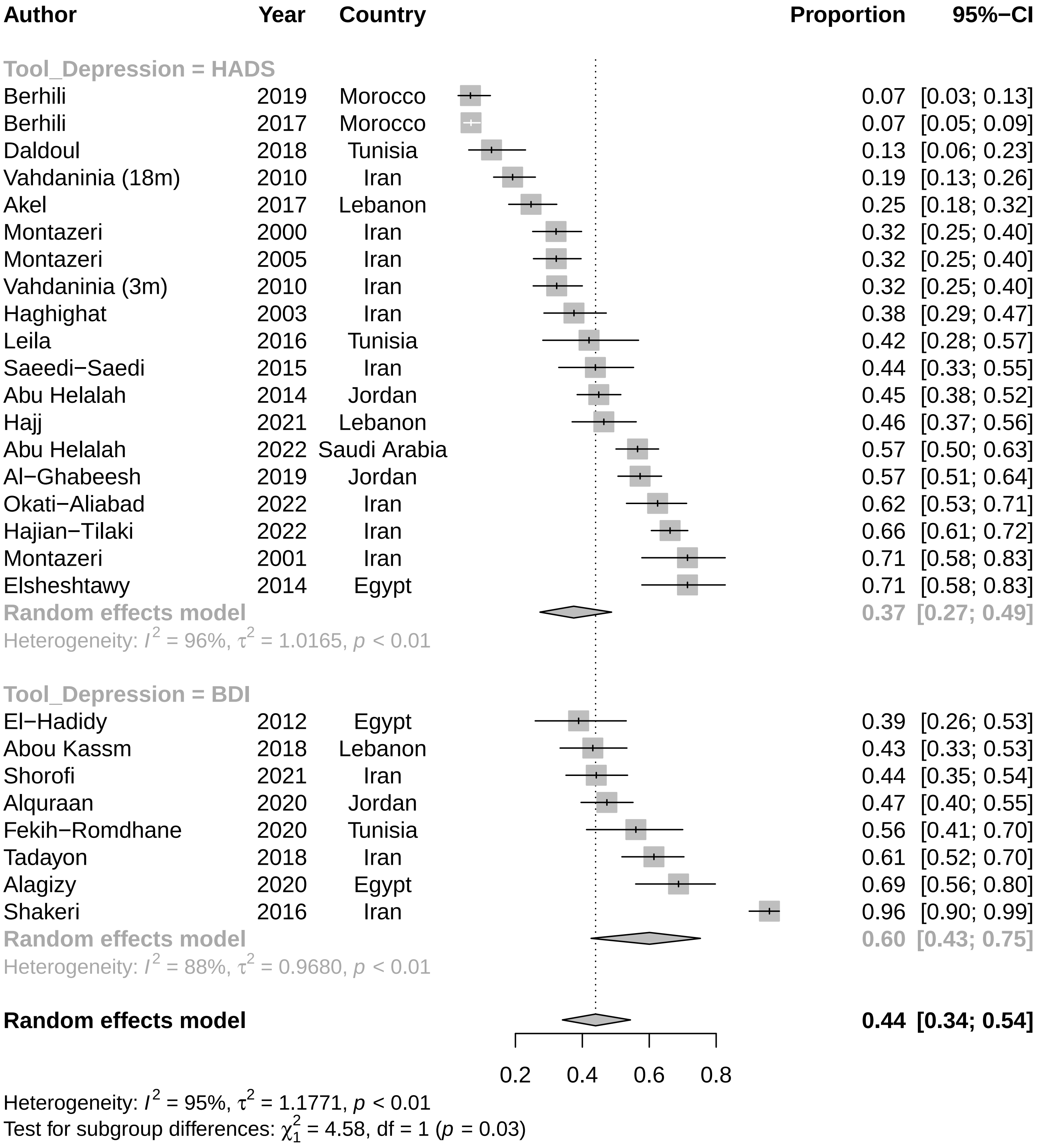

Supplementary figure 9

Author	Year	Country	Tool	Proportion	95%−CI
Montazeri	2000	Iran	HADS	0.69	[0.61; 0.76]
Montazeri	2001	Iran	HADS	0.39	[0.26; 0.53]
Haghighat	2003	Iran	HADS	0.70	[0.60; 0.78]
Montazeri	2004	Iran	SCID−I/NP	0.28	[0.23; 0.35]
Montazeri	2005	Iran	HADS	0.29	[0.22; 0.36]
Vahdaninia (3m)	2010	Iran	HADS	0.69	[0.61; 0.76]
Vahdaninia (18m)	2010	Iran	HADS	0.32	[0.25; 0.40]
El−Hadidy	2012	Egypt	HAM−A	0.30	[0.18; 0.44]
Abu Helalah	2014	Jordan	HADS	0.52	[0.46; 0.59]
Elsheshtawy	2014	Egypt	HADS	0.54	[0.40; 0.67]
Al−Zaben	2015	Saudi Arabia	HADS	0.24	[0.13; 0.39]
Leila	2016	Tunisia	HADS	0.44	[0.30; 0.59]
Berhili	2017	Morocco	HADS	0.06	[0.04; 0.08]
Akel	2017	Lebanon	HADS	0.41	[0.33; 0.50]
Daldoul	2018	Tunisia	HADS	0.21	[0.13; 0.33]
Al−Ghabeesh	2019	Jordan	HADS	0.62	[0.56; 0.69]
Berhili	2019	Morocco	HADS	0.08	[0.04; 0.15]
Farbood	2020	Iran	SAI	0.53	[0.38; 0.67]
Yektatalab	2020	Iran	SAI	0.69	[0.63; 0.75]
Alagizy	2020	Egypt	MAS	0.73	[0.61; 0.84]
Hajj	2021	Lebanon	HADS	0.56	[0.47; 0.66]
Fekih−Romdhane	2021	Tunisia	DASS−21	0.18	[0.09; 0.30]
Soqia	2022	Syria	GAD	0.36	[0.31; 0.40]
Abu Helalah	2022	Saudi Arabia	HADS	0.43	[0.37; 0.50]
Hajian−Tilaki	2022	Iran	HADS	0.78	[0.73; 0.83]
Okati−Aliabad	2022	Iran	HADS	0.60	[0.51; 0.69]
Shamieh	2022	Jordan	ESAS	0.63	[0.57; 0.69]
**Random effects model**				**0.44**	**[0.35; 0.53]**

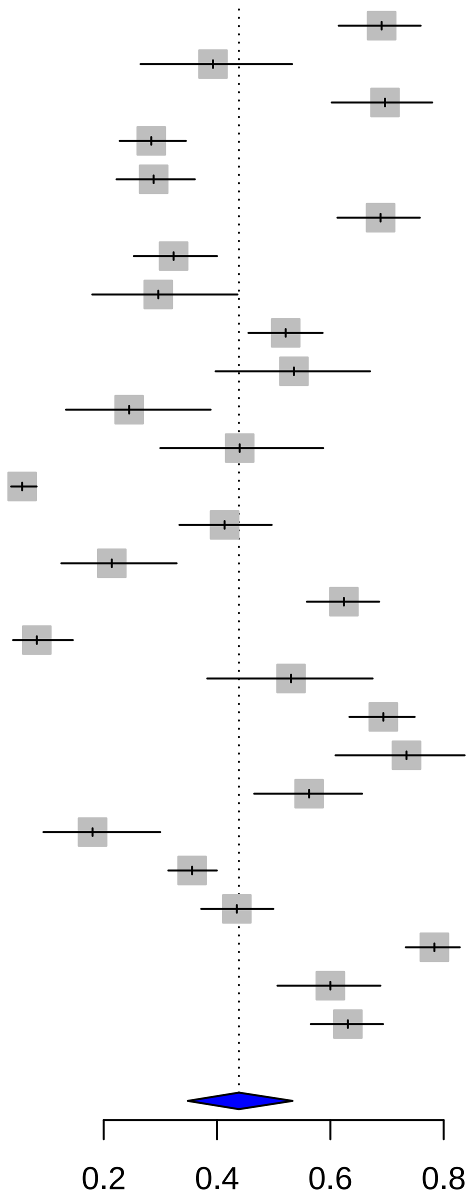

Heterogeneity: $I^2 = 96\%$, $\tau^2 = 0.9650$, $p < 0.01$

Supplementary figure 10

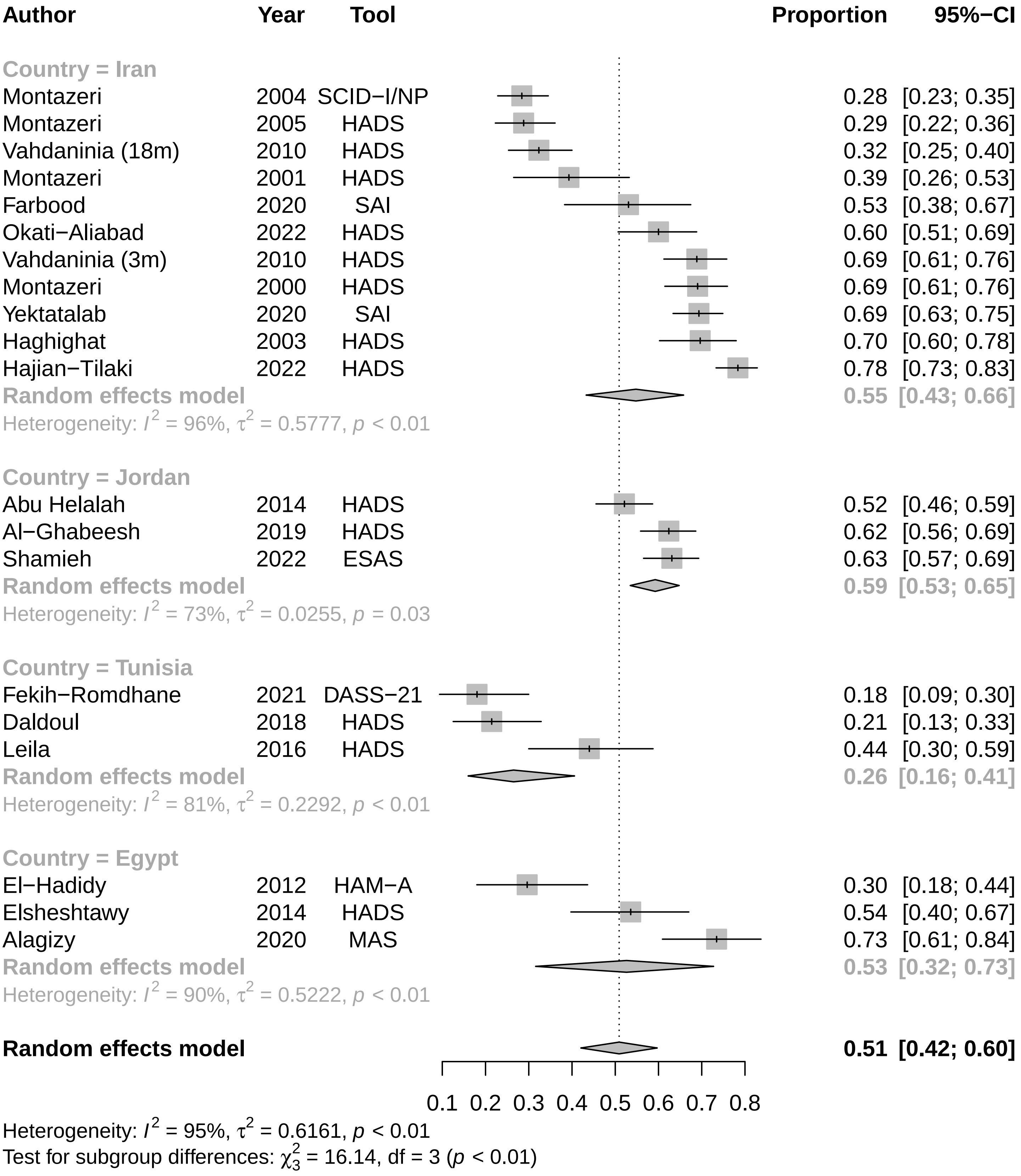

Supplementary figure 11

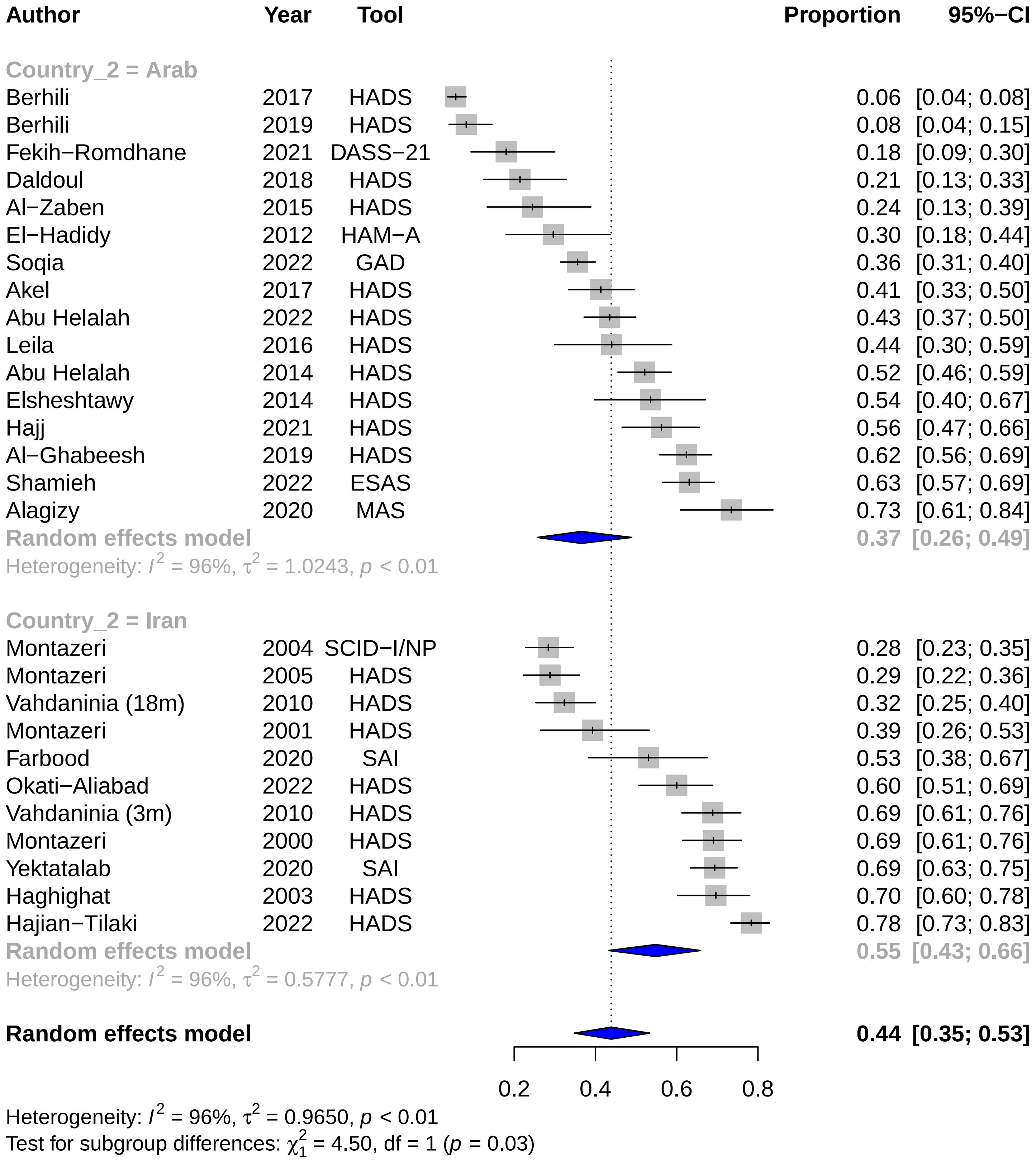

Supplementary figure 12

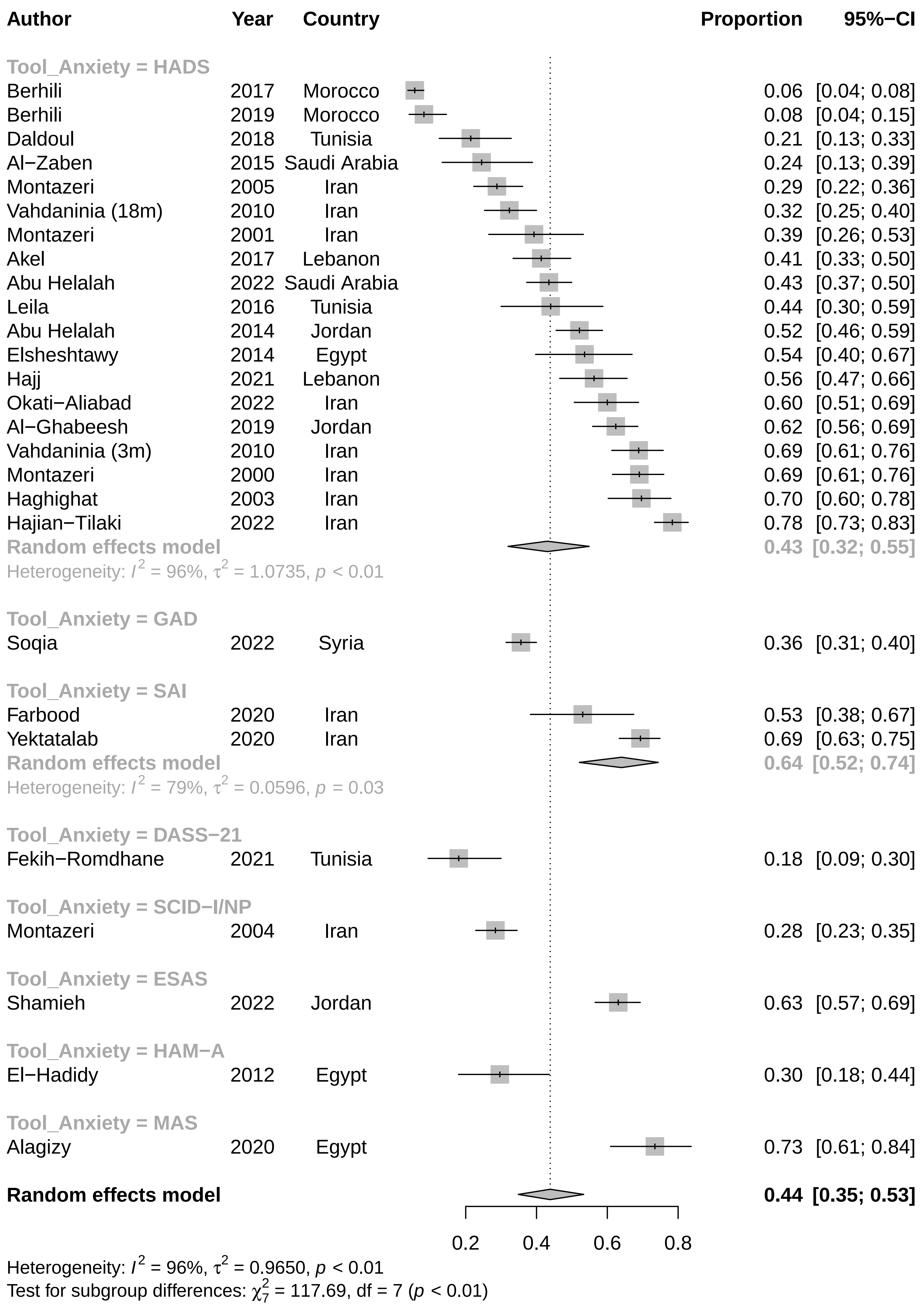

Supplementary figure 13

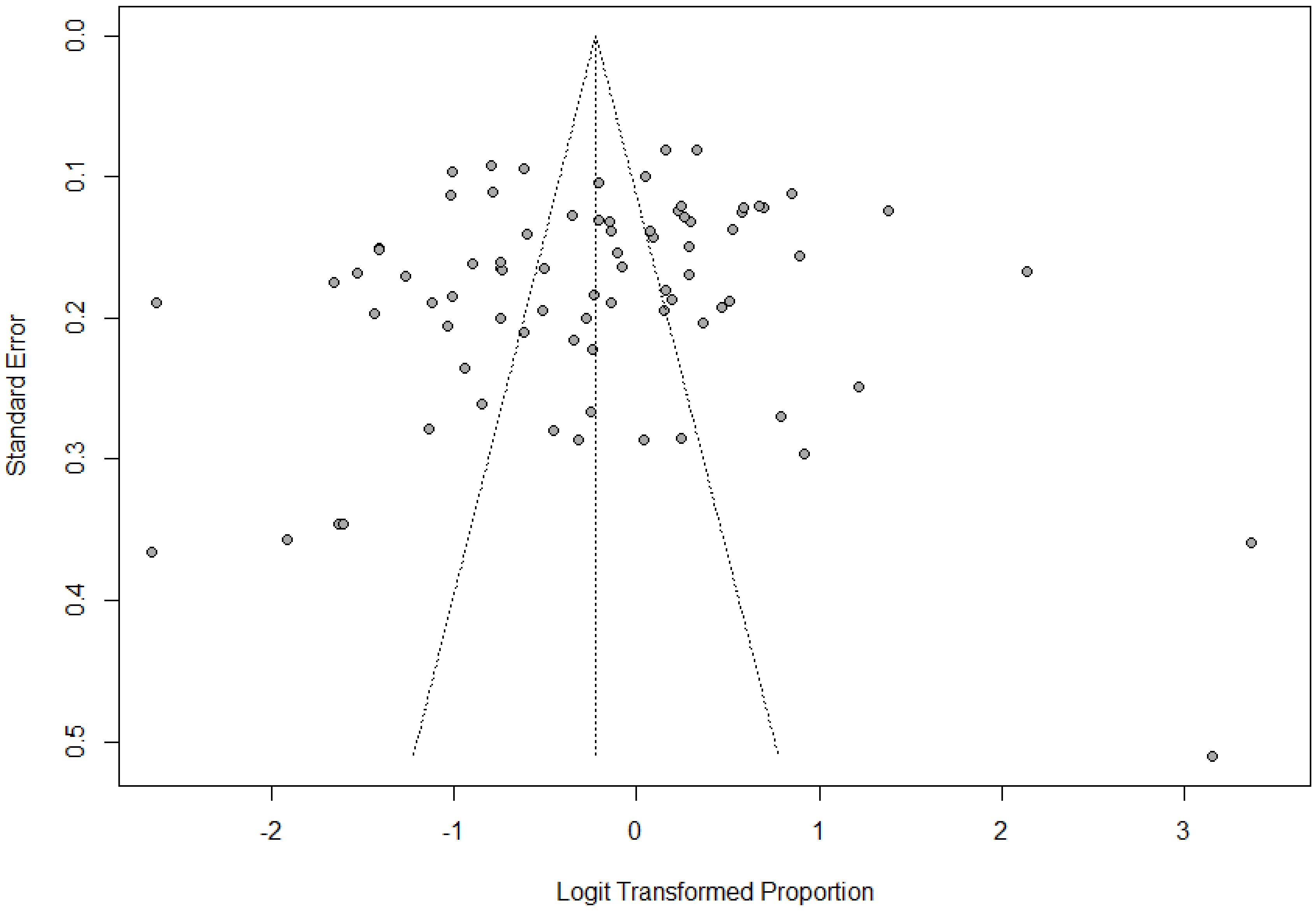

Supplementary figure 14

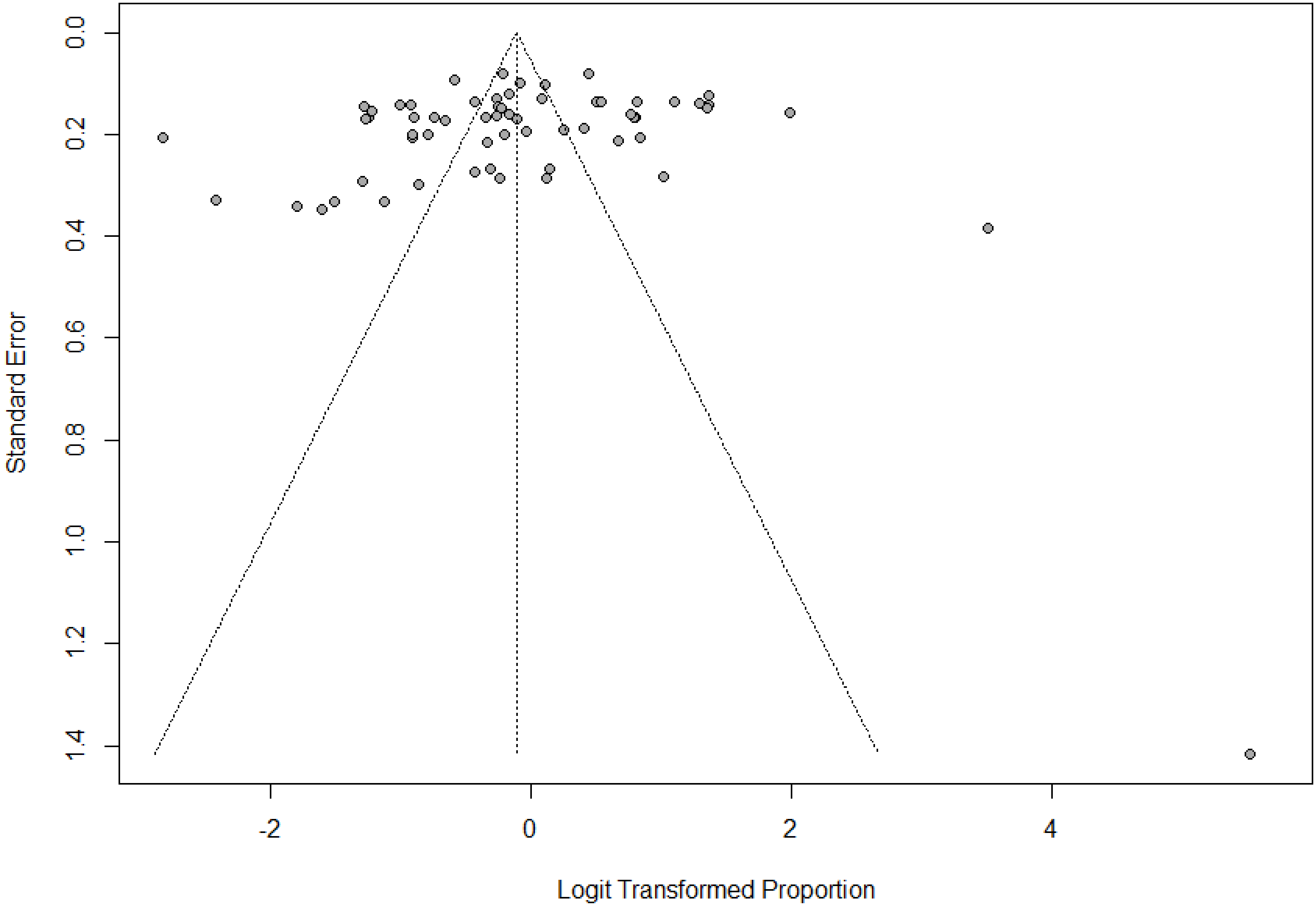

Supplement: oyae193_suppl_Supplementary_Material [file oyae193_suppl_supplementary_material.pdf]
